# Consumption of quinolones in the community, European Union/European Economic Area, 1997–2017

**DOI:** 10.1093/jac/dkab176

**Published:** 2021-08-01

**Authors:** Niels Adriaenssens, Robin Bruyndonckx, Ann Versporten, Niel Hens, Dominique L Monnet, Geert Molenberghs, Herman Goossens, Klaus Weist, Samuel Coenen, the ESAC-Net study group, Reinhild Strauss, Eline Vandael, Stefana Sabtcheva, Arjana Tambić Andrašević, Isavella Kyriakidou, Jiří Vlček, Ute Wolff Sönksen, Elviira Linask, Emmi Sarvikivi, Karima Hider-Mlynarz, Anette Zawinell, Flora Kontopidou, Mária Matuz, Gudrun Aspelund, Karen Burns, Filomena Fortinguerra, Elīna Dimiņa, Jolanta Kuklytė, Marcel Bruch, Peter Zarb, Stephanie Natsch, Hege Salvesen Blix, Anna Olczak-Pieńkowska, Ana Silva, Gabriel Adrian Popescu, Tomáš Tesař, Milan Čižman, Antonio López Navas, Vendela Bergfeldt, Berit Müller-Pebody

**Affiliations:** 1 Laboratory of Medical Microbiology, Vaccine & Infectious Disease Institute (VAXINFECTIO), University of Antwerp, Antwerp, Belgium; 2 Centre for General Practice, Department of Family Medicine & Population Health (FAMPOP), University of Antwerp, Antwerp, Belgium; 3 Interuniversity Institute for Biostatistics and statistical Bioinformatics (I-BIOSTAT), Data Science Institute, Hasselt University, Hasselt, Belgium; 4 Centre for Health Economic Research and Modelling Infectious Diseases, Vaccine & Infectious Disease Institute (VAXINFECTIO), University of Antwerp, Antwerp, Belgium; 5 Disease Programmes Unit, European Centre for Disease Prevention and Control, Stockholm, Sweden; 6 Interuniversity Institute for Biostatistics and statistical Bioinformatics (I-BIOSTAT), Catholic University of Leuven, Leuven, Belgium

## Abstract

**Objectives:**

Data on quinolone consumption in the community were collected from 30 EU/European Economic Area (EEA) countries over two decades. This article reviews temporal trends, seasonal variation, presence of change-points and changes in the composition of main subgroups of quinolones.

**Methods:**

For the period 1997–2017, data on consumption of quinolones, i.e. ATC group J01M, in the community and aggregated at the level of the active substance, were collected using the WHO ATC/DDD methodology (ATC/DDD index 2019). Consumption was expressed in DDD per 1000 inhabitants per day and in packages per 1000 inhabitants per day. Quinolone consumption was analysed by subgroups based on pharmacokinetic profile, and presented as trends, seasonal variation, presence of change-points and compositional changes.

**Results:**

In 2017, quinolone consumption in the community expressed in DDD per 1000 inhabitants per day varied by a factor of 8.2 between countries with the highest (Bulgaria) and the lowest (Norway) consumption. The second-generation quinolones accounted for >50% of quinolone consumption in most countries. Quinolone consumption significantly increased up to 2001, and did not change significantly afterwards. Seasonal variation increased significantly over time. Proportional consumption of third-generation quinolones significantly increased over time relative to that of second-generation quinolones, while proportional consumption of both third- and second-generation quinolones significantly increased relative to that of first-generation quinolones. Levofloxacin and moxifloxacin represented >40% of quinolone consumption in the community in southern EU/EEA countries.

**Conclusions:**

Quinolone consumption in the community is no longer increasing in the EU/EEA, but its seasonal variation continues to increase significantly as is the proportion of quinolones to treat respiratory infections.

## Introduction

This article presents data from the European Surveillance of Antimicrobial Consumption Network (ESAC-Net,^1^ formerly ESAC) on consumption of quinolones in the community (i.e. primary care sector) for 30 EU/European Economic Area (EEA) countries in 2017. It updates previous ESAC studies published in 2006 and 2011, and in doing so it provides updated comparable and reliable information on antibiotic consumption that can aid in fighting the global problem of antimicrobial resistance.^2^^,^^3^ In 2017, quinolones represented 9.5% of antibiotic consumption in the community.^4^ As in the previous series, quinolones were classified in three generations as introduced by Ball,^5^ based on their chemical structure and antimicrobial activity.^2^^,^^3^ The objective of this study was to analyse temporal trends, seasonal variation and the presence of change-points in quinolone consumption in the community for the period 1997–2017, as well as to analyse the composition of quinolone consumption over time.

## Methods

The methods for collecting and analysing the data are described in the introductory article of this series.^6^ In summary, data on the consumption of quinolones, i.e. quinolone antibacterials (ATC group J01M), and aggregated at the level of the active substance, were collected using the WHO ATC/DDD methodology (WHO/ATC index 2019)^6^ and expressed in DDD per 1000 inhabitants per day. In addition, where data were available, quinolone consumption was also expressed in packages per 1000 inhabitants per day. For quinolones, a classification according to chemical structure and antimicrobial activity, subdividing quinolones into three generations was used to assess quinolone consumption in the community in more detail (Table 1).^5^

**Table 1.** Clinical classification of quinolones (J01M; ATC/DDD index 2019)

First-generation		Second-generation		Third-generation	
J01MA06	**Norfloxacin** ^a^	J01MA01	Ofloxacin^a^	J01MA05	Temafloxacin^b^
J01MB01	*Rosoxacin* ^b^	J01MA02	**Ciprofloxacin** ^a^	J01MA13	*Trovafloxacin* ^b^
J01MB02	Nalidixic acid	J01MA03	Pefloxacin	J01MA14	**Moxifloxacin** ^a^
J01MB03	*Piromidic acid* ^b^	J01MA04	Enoxacin	J01MA15	*Gemifloxacin* ^b^
J01MB04	Pipemidic acid	J01MA07	Lomefloxacin	J01MA16	*Gatifloxacin* ^b^
J01MB05	*Oxolinic acid* ^b^	J01MA08	*Fleroxacin* ^b^	J01MA17	Prulifloxacin
J01MB06	*Cinoxacin*	J01MA09	*Sparfloxacin* ^b^	J01MA18	*Pazufloxacin* ^b^
J01MB07	Flumequine	J01MA10	Rufloxacin	J01MA19	*Garenoxacin* ^b^
J01MB08	*Nemonoxacin* ^c^	J01MA11	*Grepafloxacin* ^b^	J01MA21	*Sitafloxacin* ^c^
		J01MA12	**Levofloxacin** ^a^		

**Bold type** indicates that consumption was part of the top 90% of the community consumption of quinolones (J01M) in 28 EU/EEA countries in 2017; *Italic type* indicates that no consumption of this quinolone was reported in 28 EU/EEA countries in 2017.

a Consumption was part of the top 90% of the community consumption of quinolones (J01M) in 30 EU/EEA countries in 2009.

b No consumption of this quinolone was reported in 30 EU/EEA countries in 2009.

c This quinolone was not included in the ATC/DDD index in 2009 and was therefore not reported in 2009.

There are 28 unique ATC codes for quinolones in the ATC/DDD index 2019. Compared with previous descriptions of the consumption of quinolones in the community, two additional substances, i.e. sitafloxacin (J01MA21) and nemonoxacin (J01MB08) have been assigned an ATC code by the WHO (Table 1).

The evolution of the number of DDD per package over time was assessed using a linear mixed model. The temporal trend, seasonal variation and presence of change-points in quinolone consumption were assessed using a non-linear change-point mixed model fitted to quarterly data expressed in DDD per 1000 inhabitants per day from 1997 to 2017.^7^ The relative proportions of the main subgroups were assessed through a compositional data analysis modelling yearly data expressed in DDD per 1000 inhabitants per day from 1997 to 2017.^8^

## Results

An overview of consumption of quinolones (ATC J01M) in the community, expressed in DDD and packages per 1000 inhabitants per day for all participating countries between 1997 and 2017 is available as Supplementary data at *JAC* Online (Tables S1 and S2, respectively).

### Quinolone consumption in the community in 2017

In 2017, four substances accounted for 90% of quinolone consumption in the community expressed in DDD per 1000 inhabitants per day: ciprofloxacin (48.6% in 2017 compared with 50.8% in 2009), levofloxacin (28.8% in 2017 compared with 11.8% in 2009), norfloxacin (10.4% in 2017 compared with 18.2% in 2009) and moxifloxacin (7.2% in 2017 compared with 7.4% in 2009) (Table 1).

**Figure 1.**
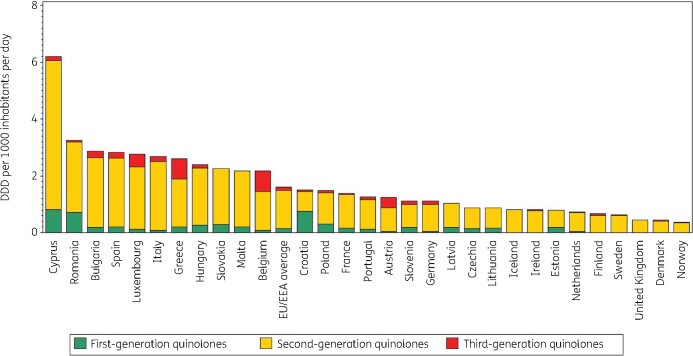
Consumption of quinolones (ATC J01M) in the community, expressed in DDD (ATC/DDD index 2019) per 1000 inhabitants per day, 30 EU/EEA countries, 2017. For Czechia, 2015 data are used. For Slovakia, 2016 data are used. For Cyprus and Romania, total care data, i.e. community and hospital sector combined, are used.

Figure 1 shows quinolone consumption, as well as the consumption of the three generations of quinolones, in the community expressed in DDD per 1000 inhabitants per day in 2017. Quinolone consumption in the community varied by a factor of 8.2 between countries with the highest (2.86 DDD per 1000 inhabitants per day in Bulgaria) and the lowest (0.35 DDD per 1000 inhabitants per day in Norway) consumption, which was higher than in 2009 (factor of 7.5, from 3.61 DDD per 1000 inhabitants per day in Italy to 0.48 DDD per 1000 inhabitants per day in the United Kingdom). We observed substantial inter-country variability in the consumption of first- and third-generation quinolones. Consumption of second-generation quinolones showed slightly less variability between countries (Table S1).

In 2017, first-generation quinolones (mostly norfloxacin) represented the most consumed quinolone subgroup only in Croatia, and represented >20% of quinolone consumption in the community in Estonia, Lithuania, Poland and Romania (total care data). Pipemidic acid was used in five countries. Among the other first-generation quinolones, nalidixic acid was only reported in Romania (total care data) and flumequine was only used in France. Second-generation quinolones were by far the most widely consumed quinolones in EU/EEA countries. Their consumption exceeded 50% (median 85%) of quinolone consumption in the community in all countries except Croatia. Ciprofloxacin was the most consumed second-generation quinolone in 24 countries; levofloxacin was the most consumed in Bulgaria, Cyprus (total care data), Hungary and Italy; and ofloxacin was the most consumed in France.

Among the third-generation quinolones, only moxifloxacin and prulifloxacin were widely consumed in EU/EEA countries during 1997–2017. Moxifloxacin was the most consumed third-generation quinolone in all countries except Italy where prulifloxacin was the most consumed. Sweden also reported limited consumption of temafloxacin.

**Figure 2.**
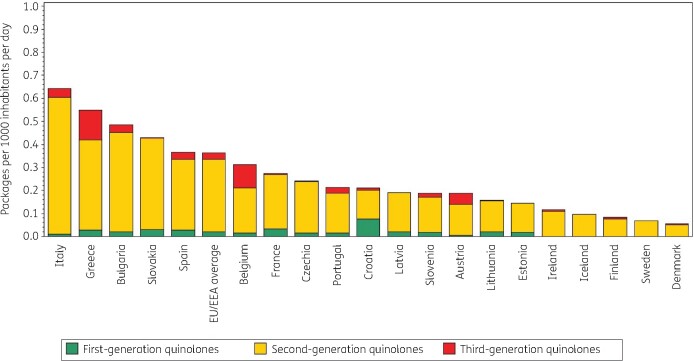
Consumption of quinolones (ATC J01M) in the community, expressed in packages per 1000 inhabitants per day, 20 EU/EEA countries, 2017. For Czechia, 2015 data are used. For Slovakia, 2016 data are used. For Cyprus and Romania, total care data, i.e. community and hospital sector combined, are used.

Figure 2 shows quinolone consumption in the community expressed in packages per 1000 inhabitants per day for 20 EU/EEA countries in 2017. Czechia ranked 13th for its quinolone consumption in DDD per 1000 inhabitants per day and 8th in packages per 1000 inhabitants per day (Table 2). The number of DDD per package ranged from 3.6 in Czechia to 9.1 in Sweden and was higher than in 2009 (from 2.2 in Italy to 8.5 in Sweden in 2009). In the EU/EEA countries, the number of DDD per package did not change significantly over time during 1997–2017.

**Table 2.** Ranking of consumption of quinolones (ATC J01M) in the community, expressed in DDDs or packages per 1000 inhabitants per day, 20 EU/EEA countries, 2017

Country	Italy	Greece	Bulgaria	Slovakia	Spain	Belgium	France	Czechia	Portugal	Croatia	Latvia	Slovenia	Austria	Lithuania	Estonia	Ireland	Iceland	Finland	Sweden	Denmark
Ranking for packages per 1000 inhabitants per day	1	2	3	4	5	6	7	8	9	10	11	12	13	14	15	16	17	18	19	20
Ranking for DDD per 1000 inhabitants per day	3	4	1	5	2	6	8	13	9	7	12	11	10	14	17	16	15	18	19	20
Number of DDD per package	4.2	4.7	5.9	5.3	7.7	7.0	5.0	3.6	5.9	7.1	5.4	5.9	6.6	5.6	5.4	7.0	8.4	8.1	9.1	7.8

For Czechia, 2015 data are used. For Slovakia, 2016 data are used. For Cyprus and Romania, total care data, i.e. community and hospital sector combined, are used.

### Longitudinal data analysis, 1997–2017

**Figure 3.**
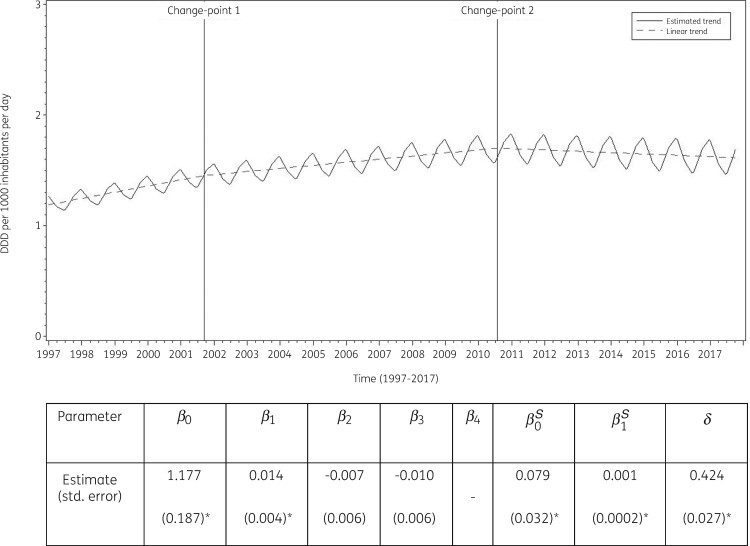
Estimated trend (solid line) and linear trend (dashed line) of consumption of quinolones (ATC J01M) in the community based on quarterly data, 25 EU/EEA countries, 1997–2017. *β_0_*, predicted consumption in the first quarter of 1997; *β_1_*, predicted increase (if positive)/decrease (if negative) in consumption per quarter; *β_2_*, predicted difference in slope after versus before the first change-point; *β_3_*, predicted difference in slope after versus before the second change-point; *β_4_*, predicted difference in slope after versus before the third change-point; *β_0_^S^*, predicted amplitude of the upward winter and downward summer peak in consumption; *β_1_^S^*, predicted increase (if positive)/decrease (if negative) of the amplitude of the upward winter and downward summer peak in consumption per quarter; *δ*, shift in timing of the upward winter and downward summer peak from one year to another. An asterisk indicates that the result was statistically significant at significance level 0.05.

The best fit was obtained for a model including two change-points: one in the third quarter of 2001 and another in the third quarter of 2010. The final model fits the observed data well (Figure S1). The longitudinal data analysis estimated an average quinolone consumption in the EU/EEA of 1.177 (SE 0.187) DDD per 1000 inhabitants per day in the first quarter of 1997. In addition, the analysis showed a significant seasonal variation with an amplitude of 0.079 (SE 0.032) DDD per 1000 inhabitants per day, which increased significantly over time (+0.001, SE 0.0002, DDD per 1000 inhabitants per day per quarter). Quinolone consumption significantly increased (+0.014, SE 0.005, DDD per 1000 inhabitants per day per quarter) between 1997 and the third quarter of 2001. After this first change-point, no significant change was observed (+0.007, SE 0.006, DDD per 1000 inhabitants per day per quarter until the third quarter of 2010, after which there was a decrease of −0.003, SE 0.008, DDD per 1000 inhabitants per day per quarter) (Figure 3).

Based on the fitted model, community quinolone consumption in 1997 was significantly higher than average in Belgium, Greece, Italy, Luxembourg, Portugal and Spain, and significantly lower than average in Denmark, Finland, Hungary, Iceland, Lithuania and the United Kingdom (observed profiles shown in Figure S2 and S3). The seasonal variation was significantly larger than average in Belgium, Germany, Hungary, Italy, Luxembourg, Portugal and Spain, and significantly smaller than average in Cyprus (total care data), Denmark, Estonia, Finland, Iceland, Ireland, the Netherlands and the United Kingdom. The increase in quinolone consumption between 1997 and the third quarter of 2001 was significantly larger than average in Belgium and Hungary. The increase between the last quarter of 2001 and the third quarter of 2010 was significantly larger than average in Cyprus (total care data), Italy, Lithuania and Slovakia. The decrease in quinolone consumption between the last quarter of 2010 and 2017 was significantly larger than average in Italy and Portugal.

Table S1 provides an overview of quinolone consumption in the community in the participating countries between 1997 and 2017. Quinolone consumption decreased in several countries. This decrease was the highest in Portugal, but also considerable in Italy and France. The largest increases in quinolone consumption in the community were observed for Cyprus, Romania, Bulgaria, Hungary and Malta. The seasonal variation in quinolone consumption is shown in Figures S2 and S3. In all countries except Belgium and Italy, the mean quinolone consumption in the first and fourth (winter) quarters did not exceed the mean consumption in the second and third (summer) quarters by >20%. The high seasonal variation in Belgium and Italy resulted from the relatively frequent consumption of third-generation quinolones in the winter quarters. Moxifloxacin and levofloxacin showed the highest seasonal variation. Moxifloxacin consumption was >50% higher in winter quarters compared with summer quarters in Austria, Belgium, Estonia, Italy, Latvia, Luxembourg and Portugal. Levofloxacin consumption was >50% higher in winter quarters compared with summer quarters in Austria, Belgium, Croatia, Denmark, Estonia and Finland.

### Compositional data analysis, 1997–2009

The proportional consumption of third-generation quinolones significantly increased over time relative to that of second-generation quinolones, whilst the consumption of both second- and third-generation quinolones significantly increased relative to that of first-generation quinolones (Table 3).

**Table 3.** Change in the composition of the consumption of quinolones (ATC J01M) in the community, expressed in DDD (ATC/DDD index 2019) per 1000 inhabitants per day, 30 EU/EEA countries, as a function of time during 1997–2017

	1GQ	2GQ	3GQ
1GQ		**−0.1230**	**−0.1865**
2GQ	**0.1230**		**−0.0634**
3GQ	**0.1865**	**0.0634**	

Values are estimated changes in the log ratio of the row versus column subgroup of antibiotics with increasing time. Bold type indicates a statistically significant effect; positive values represent an increase and negative values represent a decrease.

1GQ, first-generation quinolones; 2GQ, second-generation quinolones; 3GQ, third-generation quinolones.

Trends of proportional consumption in individual countries are shown in Figure S4. When comparing the composition of quinolone consumption in 2017 with that in 2009, the proportion of first-generation quinolones decreased for most of the participating countries with the largest decreases observed for Czechia (−28.69%; 2015 data), Cyprus (−3.48%; total care data), the Netherlands (−21.07%), Poland (−19.45%) and France (−15.51%). These decreases can mainly be explained by decreasing norfloxacin consumption. For Italy and Lithuania, the decrease also resulted from decreasing consumption of pipemidic acid. In most countries, the decrease in the proportion of first-generation quinolones was matched by a similar increase in the consumption of second-generation quinolones. The largest increases in the proportion of second-generation quinolones were observed for Czechia (+28.16%; 2015 data), Cyprus (+25.09%; total care data), the Netherlands (+21.80%) and France (+21.37%). The proportion of third-generation quinolones showed both increases and decreases, with the largest increases observed for Greece (+18.67%), Austria (+10.86%) and Poland (+6.42%), and the largest decreases for Portugal (−11.39%), Malta (−9.25%) and Italy (−8.48%).

The proportional consumption of levofloxacin and moxifloxacin combined out of quinolone consumption in the community is shown in Figure S5. In most countries, this proportion has been increasing since they were introduced. In 2017, the proportion of levofloxacin and moxifloxacin combined represented >50% of quinolone consumption in the community in Bulgaria, Cyprus (total care data), Hungary and Italy, >40% in Belgium, Malta and Spain, and >30% in Austria, Finland, Germany, Greece, Portugal and Romania (total care data).

## Discussion

This study describes consumption of quinolones in the community in the EU/EEA, which has been described as one of the fastest growing antibiotic classes since the start of surveillance of antimicrobial consumption in Europe in 1997.^2^^,^^3^ The longitudinal data analysis shows this increase has in general stopped. Yet, variation between countries has increased, with Bulgaria having the highest quinolone consumption in the community (mainly levofloxacin) in 2017 and some countries, mainly in Southern and Eastern Europe, showing a substantial increase in quinolone consumption in the community compared with 2009.

In 2017, the consumption of quinolones out of all antibacterials for systemic use (J01) ranged from 2.46% in Norway to 21.49% in Cyprus.^4^ In European countries that are not part of the ESAC-Net but covered by the WHO Europe Antimicrobial Medicines Consumption (AMC) Network, a similar range was observed, i.e. from 0.5% (Kyrgyzstan) to 17.8% (Kazakhstan) of total, i.e. community and hospital sector combined, consumption.^9^

Ciprofloxacin remained the most consumed quinolone in most countries. Yet, an emerging trend to consume more levofloxacin and moxifloxacin, mainly in countries with a high quinolone consumption, should be noted (Figure S5). Moreover, seasonal variation increased in the EU/EEA over time during 1997–2017 suggesting increasing inappropriate use of quinolones to treat respiratory tract infections. Quinolones must be considered as broad-spectrum antibiotics and are not recommended as first-line antibiotics for the treatment of respiratory tract infections in the community. The potential small superiority of third-generation quinolones, as compared with penicillin and macrolides, for the treatment of respiratory tract infections should be balanced against concerns of selection pressure and cost.^10^ In addition, ciprofloxacin is contraindicated for the treatment of community-acquired pneumonia because it is not active against *Streptococcus pneumoniae*.^11^ Quinolone consumption in DDD per 1000 inhabitants as well as seasonal variation of quinolone consumption are among the final set of 12 ESAC drug-specific quality indicators for outpatient antibiotic consumption in Europe.^12^ The 2017 values for the drug-specific quality indicators are reported in an accompanying article.^13^ The ESAC disease-specific quality indicators for outpatient antibiotic use also focus on quinolone consumption, with an upper limit of 5% quinolone prescriptions in patients being prescribed an antibiotic for any of the seven listed indications.^14^ In Belgium for example, the National Institute for Health and Disability Insurance (NIHDI) restricted the prescription of quinolones to certain diagnoses (mainly urinary tract) and conditions.^15^^,^^16^ A patient will only receive reimbursement for a quinolone prescription in case of adherence to these restrictions. It is too soon to evaluate this policy change with the available ESAC-Net data, but preliminary results show that between 2017 and 2018 the consumption of fluoroquinolones in the community declined by 46% when based on reimbursement data but only by 25% based on sales data.^17^ This suggests that such policy interventions need careful implementation as prescribing and choice of an antibiotic according to the guidelines is an individual doctor’s decision.

Based on a recommendation from EMA and following the suspension of the marketing authorization of medicines containing cinoxacin, flumequine, nalidixic acid, and pipemidic acid, the European Commission decided to restrict the use of the remaining fluoroquinolones because of the risk of disabling and potentially permanent side-effects. According to the EMA’s recommendation, fluoroquinolones should not be used (a) to treat infections that might get better without treatment or infections that are not severe (such as throat infections); (b) to treat non-bacterial infections, e.g. non-bacterial (chronic) prostatitis; (c) to prevent traveller’s diarrhoea or recurring lower urinary tract infections (urine infections that do not extend beyond the bladder); (d) to treat mild or moderate bacterial infections unless other antibacterial medicines commonly recommended for these infections cannot be used.^18^

As quinolone consumption should be restricted and mainly reserved for well-defined indications, the high consumption and seasonal variation of quinolones in the community observed in some countries probably indicates non-adherence to prescribing guidelines. From a public health perspective, this is an important consideration, as excessive and inappropriate use of quinolones is associated with the development of quinolone resistance, requires more resources and exposes patients to the additional risk of side effects.^19^^,^^20^

All quinolones (ATC J01M) are listed as Watch group antibiotics in the 2019 WHO Access, Watch or Reserve (AWaRe) classification list.^21^ The continuous monitoring of quinolone consumption in the community can help to assess the impact of future interventions promoting better use of these antibiotics.

For a more-detailed discussion on the limitations of the collected data, we refer to the article on antibacterials for systemic use, included in this series.^6^ For a discussion on the limitations of the statistical approach used in this study and potential explanations for the common change-points detected through these analyses, we refer to the tutorial included in this series.^7^

In conclusion, even though community quinolone consumption in the EU/EEA stopped increasing, there is still substantial seasonal variation, which suggests inappropriate prescribing of quinolones in the community in many countries. This could be a target for future awareness campaigns for more-prudent use of antibiotics.

## Supplementary Material

dkab176_Supplementary_Data

## References

[1] European Centre for Disease Prevention and Control (ECDC). European Surveillance of Antimicrobial Consumption Network (ESAC-Net). 2020.

[2] Ferech M, Coenen S, Malhotra-Kumar S et al European Surveillance of Antimicrobial Consumption (ESAC): outpatient quinolone use in Europe. J Antimicrob Chemother 2006; 58: 423–7. 16735418 10.1093/jac/dkl183

[3] Adriaenssens N, Coenen S, Versporten A et al European Surveillance on Antimicrobial Consumption: outpatient quinolone use in Europe (1997–2009). J Antimicrob Chemother 2011; 66 Suppl 6: vi47–56. 22096066 10.1093/jac/dkr457

[4] Bruyndonckx R, Adriaenssens N, Versporten A et al Consumption of antibiotics in the community, European Union/European Economic Area, 1997–2017. J Antimicrob Chemother 2021; 76 Suppl 2: ii7–ii13. 34312654 10.1093/jac/dkab172 PMC8314117

[5] Ball P. Quinolone generations: natural history or natural selection? J Antimicrob Chemother 2000; 46: 17–24. 10997595 10.1093/oxfordjournals.jac.a020889

[6] Bruyndonckx R, Adriaenssens N, Versporten A et al Consumption of antibiotics in the community, European Union/European Economic Area, 1997–2017: data collection, management and analysis. J Antimicrob Chemother 2021; 76 Suppl 2: ii2–ii6. 34312651 10.1093/jac/dkab171 PMC8314094

[7] Bruyndonckx R, Coenen S, Adriaenssens N et al Analysing the trend over time of antibiotic consumption in the community: a tutorial on the detection of common change-points. J Antimicrob Chemother 2021; 76 Suppl 2: ii79–ii85. 34312655 10.1093/jac/dkab180 PMC8314099

[8] Faes C, Molenberghs G, Hens N et al Analysing the composition of outpatient antibiotic use: a tutorial on compositional data analysis. J Antimicrob Chemother 2011; 66 Suppl 6: vi89–94. 22096070 10.1093/jac/dkr461

[9] WHO Regional Office for Europe Antimicrobial Medicines Consumption (AMC) Network. AMC data 2011–2017. 2020.

[10] Salkind AR, Cuddy PG, Foxworth JW. Fluoroquinolone treatment of community-acquired pneumonia: a meta-analysis. Ann Pharmacother 2002; 36: 1938–43. 12452758 10.1345/aph.1C167

[11] Woodhead M, Blasi F, Ewig S et al Guidelines for the management of adult lower respiratory tract infections—full version. Clin Microbiol Infect 2011; 17: E1–59. 10.1111/j.1469-0691.2011.03672.x PMC7128977 21951385

[12] Coenen S, Ferech M, Haaijer-Ruskamp FM et al European Surveillance of Antimicrobial Consumption (ESAC): quality indicators for outpatient antibiotic use in Europe. Qual Saf Health Care 2007; 16: 440–5. 18055888 10.1136/qshc.2006.021121 PMC2653179

[13] Adriaenssens N, Bruyndonckx R, Versporten A et al Quality appraisal of antibiotic consumption in the community, European Union/European Economic Area, 2009 and 2017. J Antimicrob Chemother 2021; 76 Suppl 2: ii60–ii67. 34312656 10.1093/jac/dkab178 PMC8314110

[14] Adriaenssens N, Coenen S, Tonkin-Crine S et al European Surveillance of Antimicrobial Consumption (ESAC): disease-specific quality indicators for outpatient antibiotic prescribing. BMJ Qual Saf 2011; 20: 764–72. 10.1136/bmjqs.2010.049049 21441602

[15] Bruyndonckx R, Coenen S, Hens N et al Antibiotic use and resistance in Belgium: the impact of two decades of multi-faceted campaigning. Acta Clin Belg 2021; **76**: 280–8. 10.1080/17843286.2020.1721135 32024450

[16] National institute for health and disability insurance (NIHDI). Antibiotica die tot de klasse van de (fluoro)chinolonen behoren: terugbetaling vanaf 1 mei 2018 [Antibiotics belonging to the class of (fluoro)quinolones: reimbursement starting 1 May 2018]. https://www.riziv.fgov.be/nl/themas/kost-terugbetaling/door-ziekenfonds/geneesmiddel-gezondheidsproduct/terugbetalen/specialiteiten/wijzigingen/Paginas/antibiotica-fluoro-chinolonen.aspx.

[17] ECDC Country report: Antimicrobial consumption in Belgium, 2018. https://www.ecdc.europa.eu/en/antimicrobial-consumption/database/country-overview.

[18] European Medicines Agency (EMA). Quinolone- and fluoroquinolone-containing medicinal products. Amsterdam, 2020. https://www.ema.europa.eu/en/medicines/human/referrals/quinolone-fluoroquinolone-containing-medicinal-products.

[19] Stewardson AJ, Vervoort J, Adriaenssens N et al Effect of outpatient antibiotics for urinary tract infections on antimicrobial resistance among commensal Enterobacteriaceae: a multinational prospective cohort study. Clin Microbiol Infect 2018; 24: 972–9. 29331548 10.1016/j.cmi.2017.12.026

[20] Megraud F, Coenen S, Versporten A et al *Helicobacter pylori* resistance to antibiotics in Europe and its relationship to antibiotic consumption. Gut 2013; 62: 34–42. 22580412 10.1136/gutjnl-2012-302254

[21] WHO. AWaRe Classification antibiotics. https://www.who.int/news/item/01-10-2019-who-releases-the-2019-aware-classification-antibiotics.

