# Supplementary material for: Consumption of quinolones in the community, European Union/European Economic Area, 1997–2017
Source: J Antimicrob Chemother. 2021 Aug 1;76(Suppl 2):ii37–44. doi: 10.1093/jac/dkab176 (PMC8314103; doi:10.1093/jac/dkab176)
Supplement: dkab176_Supplementary_Data [file dkab176_supplementary_data.docx]

**Supplementary data**

**Table S1. Consumption of quinolones (J01M) in the community, expressed in DDD (ATC/DDD index 2019) per 1000 inhabitants per day, 30 EU/EEA countries, 1997-2017.**

| **Country** | **1997** | **1998** | **1999** | **2000** | **2001** | **2002** | **2003** | **2004** | **2005** | **2006** | **2007** | **2008** | **2009** | **2010** | **2011** | **2012** | **2013** | **2014** | **2015** | **2016** | **2017** |
| --- | --- | --- | --- | --- | --- | --- | --- | --- | --- | --- | --- | --- | --- | --- | --- | --- | --- | --- | --- | --- | --- |
| **Austria** | **-** | **1.05** | **1.14** | **1.25** | **1.35** | **1.39** | **1.32** | **1.50** | **1.42** | **1.41** | **1.43** | **1.31** | **1.33** | **1.36** | **1.29** | **1.30** | **1.47** | **1.30** | **1.31** | **1.20** | **1.23** |
| 1GQ | **-** | 0.42 | 0.41 | 0.39 | 0.37 | 0.35 | 0.33 | 0.27 | 0.24 | 0.22 | 0.20 | 0.18 | 0.17 | 0.15 | 0.13 | 0.12 | 0.10 | 0.08 | 0.07 | 0.04 | 0.03 |
| 2GQ | **-** | 0.63 | 0.72 | 0.83 | 0.85 | 0.89 | 0.81 | 1.00 | 0.91 | 0.92 | 0.94 | 0.91 | 0.93 | 0.93 | 0.88 | 0.89 | 1.02 | 0.90 | 0.88 | 0.83 | 0.85 |
| 3GQ | **-** | <0.01 | <0.01 | 0.04 | 0.12 | 0.15 | 0.19 | 0.22 | 0.27 | 0.26 | 0.29 | 0.22 | 0.23 | 0.28 | 0.28 | 0.29 | 0.35 | 0.31 | 0.37 | 0.33 | 0.35 |
| **Belgium** | **1.79** | **1.88** | **2.10** | **2.27** | **2.61** | **2.68** | **2.73** | **2.46** | **2.47** | **2.36** | **2.27** | **2.42** | **2.61** | **2.69** | **2.73** | **2.77** | **2.64** | **2.55** | **2.57** | **2.40** | **2.17** |
| 1GQ | 0.83 | 0.75 | 0.65 | 0.56 | 0.51 | 0.45 | 0.42 | 0.38 | 0.35 | 0.31 | 0.28 | 0.28 | 0.26 | 0.24 | 0.22 | 0.20 | 0.17 | 0.14 | 0.12 | 0.10 | 0.08 |
| 2GQ | 0.97 | 1.13 | 1.45 | 1.71 | 2.10 | 1.86 | 1.64 | 1.52 | 1.50 | 1.45 | 1.40 | 1.56 | 1.57 | 1.57 | 1.55 | 1.54 | 1.49 | 1.50 | 1.49 | 1.44 | 1.36 |
| 3GQ | **-** | **-** | **-** | **-** | **-** | 0.38 | 0.67 | 0.56 | 0.62 | 0.60 | 0.59 | 0.58 | 0.78 | 0.88 | 0.96 | 1.03 | 0.99 | 0.92 | 0.96 | 0.86 | 0.73 |
| **Bulgaria** | **-** | **-** | ***0.03*** | ***1.24*** | ***1.60*** | ***1.35*** | ***0.44*** | ***1.61*** | ***2.28*** | **1.81** | **1.95** | **2.08** | **1.97** | **1.99** | **2.26** | **2.40** | **2.52** | **2.87** | **2.83** | **2.78** | **2.86** |
| 1GQ | **-** | **-** | *0.01* | *0.01* | *0.03* | *0.03* | *0.05* | *0.12* | *0.16* | 0.33 | 0.34 | 0.36 | 0.32 | 0.31 | 0.28 | 0.24 | 0.22 | 0.23 | 0.20 | 0.24 | 0.19 |
| 2GQ | **-** | **-** | *0.02* | *1.22* | *1.57* | *1.32* | *0.37* | *1.47* | *2.09* | 1.46 | 1.58 | 1.67 | 1.58 | 1.60 | 1.89 | 2.09 | 2.23 | 2.56 | 2.48 | 2.36 | 2.46 |
| 3GQ | **-** | **-** | ***-*** | *<0.01* | *<0.01* | ***-*** | *0.02* | *0.02* | *0.04* | 0.02 | 0.03 | 0.05 | 0.07 | 0.08 | 0.08 | 0.07 | 0.06 | 0.08 | 0.14 | 0.18 | 0.21 |
| **Croatia** | **-** | **-** | **-** | **-** | **1.37** | **1.58** | **1.56** | **1.51** | **1.60** | **1.61** | **1.45** | **1.49** | **1.41** | **1.31** | **1.32** | **1.49** | **1.47** | **1.50** | **1.50** | **1.49** | **1.50** |
| 1GQ | **-** | **-** | **-** | **-** | 1.24 | 1.40 | 1.33 | 1.26 | 1.27 | 1.21 | 0.95 | 0.88 | 0.78 | 0.75 | 0.84 | 0.90 | 0.87 | 0.84 | 0.79 | 0.76 | 0.75 |
| 2GQ | **-** | **-** | **-** | **-** | 0.14 | 0.15 | 0.18 | 0.18 | 0.23 | 0.31 | 0.41 | 0.50 | 0.52 | 0.46 | 0.40 | 0.51 | 0.55 | 0.60 | 0.65 | 0.68 | 0.70 |
| 3GQ | **-** | **-** | **-** | **-** | **-** | 0.02 | 0.05 | 0.06 | 0.09 | 0.09 | 0.09 | 0.11 | 0.10 | 0.10 | 0.08 | 0.07 | 0.06 | 0.05 | 0.05 | 0.05 | 0.05 |
| **Cyprus** | **-** | **-** | **-** | **-** | **-** | **-** | **-** | **-** | **-** | ***3.83*** | ***3.77*** | ***4.40*** | ***4.10*** | ***4.07*** | ***3.77*** | ***3.51*** | ***3.98*** | ***3.22*** | ***4.56*** | ***7.02*** | ***6.21*** |
| 1GQ | **-** | **-** | **-** | **-** | **-** | **-** | **-** | **-** | **-** | *1.70* | *1.57* | *1.72* | *1.50* | *1.39* | *1.27* | *1.18* | *1.04* | *0.88* | *0.88* | *0.87* | *0.81* |
| 2GQ | **-** | **-** | **-** | **-** | **-** | **-** | **-** | **-** | **-** | *2.11* | *2.06* | *2.55* | *2.44* | *2.54* | *2.31* | *2.19* | *2.78* | *2.19* | *3.49* | *5.99* | *5.24* |
| 3GQ | **-** | **-** | **-** | **-** | **-** | **-** | **-** | **-** | **-** | *0.02* | *0.14* | *0.13* | *0.17* | *0.14* | *0.18* | *0.15* | *0.17* | *0.15* | *0.19* | *0.15* | *0.16* |
| **Czechia** | **-** | **0.97** | **1.08** | **-** | **-** | **-** | **1.09** | **1.27** | **1.37** | **1.15** | **1.20** | **1.21** | **1.24** | **1.19** | **1.11** | **1.03** | **0.85** | **0.88** | **0.88** | **-** | **-** |
| 1GQ | **-** | 0.69 | 0.66 | **-** | **-** | **-** | 0.53 | 0.67 | 0.71 | 0.57 | 0.59 | 0.56 | 0.55 | 0.54 | 0.49 | 0.43 | 0.17 | 0.15 | 0.14 | **-** | **-** |
| 2GQ | **-** | 0.28 | 0.42 | **-** | **-** | **-** | 0.57 | 0.60 | 0.66 | 0.58 | 0.61 | 0.65 | 0.69 | 0.65 | 0.61 | 0.60 | 0.68 | 0.73 | 0.73 | **-** | **-** |
| 3GQ | **-** | **-** | **-** | **-** | **-** | **-** | **-** | <0.01 | **-** | **-** | **-** | **-** | **-** | <0.01 | <0.01 | <0.01 | <0.01 | <0.01 | <0.01 | **-** | **-** |
| **Denmark** | **0.22** | **0.24** | **0.20** | **0.15** | **0.17** | **0.18** | **0.25** | **0.28** | **0.32** | **0.37** | **0.44** | **0.52** | **0.52** | **0.51** | **0.57** | **0.55** | **0.52** | **0.50** | **0.49** | **0.48** | **0.44** |
| 1GQ | <0.01 | <0.01 | <0.01 | <0.01 | <0.01 | <0.01 | <0.01 | **-** | **-** | **-** | **-** | **-** | **-** | **-** | **-** | **-** | **-** | **-** | **-** | **-** | **-** |
| 2GQ | 0.22 | 0.23 | 0.20 | 0.15 | 0.17 | 0.17 | 0.24 | 0.28 | 0.32 | 0.36 | 0.43 | 0.50 | 0.50 | 0.50 | 0.55 | 0.53 | 0.49 | 0.47 | 0.46 | 0.44 | 0.41 |
| 3GQ | **-** | **-** | **-** | **-** | <0.01 | <0.01 | <0.01 | <0.01 | 0.01 | 0.01 | 0.01 | 0.02 | 0.02 | 0.01 | 0.02 | 0.03 | 0.02 | 0.02 | 0.03 | 0.04 | 0.04 |
| Country, community consumption of quinolones (J01M); 1GQ, consumption of first-generation quinolones; 2GQ, consumption of second-generation quinolones; 3GQ, consumption of third-generation  quinolones; **-**, no consumption reported; Numbers reported in *italic* are total care data, i.e. community and hospital sector combined; ^a^Data for Romania have a coverage in 2009 limited to 30-40%; ^b^Data for Spain include private prescriptions from 2016 onwards. | | | | | | | | | | | | | | | | | | | | | |
| **Estonia** | **-** | **-** | **-** | **-** | ***0.82*** | **0.55** | **0.62** | **0.70** | **0.75** | **0.82** | **0.87** | **0.88** | **0.79** | **0.82** | **0.85** | **0.85** | **0.89** | **0.90** | **0.92** | **0.85** | **0.79** |
| 1GQ | **-** | **-** | **-** | **-** | *0.16* | 0.16 | 0.17 | 0.20 | 0.23 | 0.27 | 0.29 | 0.30 | 0.27 | 0.27 | 0.26 | 0.25 | 0.26 | 0.24 | 0.22 | 0.19 | 0.18 |
| 2GQ | **-** | **-** | **-** | **-** | *0.66* | 0.39 | 0.45 | 0.50 | 0.52 | 0.55 | 0.58 | 0.58 | 0.51 | 0.55 | 0.59 | 0.60 | 0.63 | 0.66 | 0.69 | 0.65 | 0.60 |
| 3GQ | **-** | **-** | **-** | **-** | ***-*** | **-** | **-** | **-** | **-** | **-** | <0.01 | <0.01 | <0.01 | <0.01 | <0.01 | <0.01 | <0.01 | <0.01 | 0.01 | <0.01 | <0.01 |
| **Finland** | **0.65** | **0.64** | **0.64** | **0.72** | **0.83** | **0.90** | **0.84** | **0.83** | **0.83** | **0.83** | **0.90** | **0.82** | **0.87** | **0.88** | **0.95** | **0.89** | **0.84** | **0.82** | **0.73** | **0.74** | **0.67** |
| 1GQ | 0.27 | 0.25 | 0.15 | 0.15 | 0.16 | 0.16 | 0.17 | 0.17 | 0.15 | 0.14 | 0.14 | 0.11 | 0.11 | 0.11 | 0.10 | 0.08 | 0.07 | 0.06 | <0.01 | **-** | **-** |
| 2GQ | 0.38 | 0.39 | 0.49 | 0.58 | 0.67 | 0.73 | 0.66 | 0.64 | 0.65 | 0.65 | 0.69 | 0.64 | 0.70 | 0.72 | 0.77 | 0.74 | 0.71 | 0.70 | 0.67 | 0.67 | 0.60 |
| 3GQ | **-** | <0.01 | <0.01 | <0.01 | 0.01 | 0.01 | 0.01 | 0.03 | 0.03 | 0.04 | 0.07 | 0.07 | 0.07 | 0.06 | 0.07 | 0.07 | 0.06 | 0.06 | 0.06 | 0.07 | 0.07 |
| **France** | **1.83** | **1.85** | **1.90** | **2.14** | **2.34** | **2.08** | **2.04** | **2.08** | **2.17** | **2.18** | **2.19** | **2.09** | **2.00** | **2.00** | **1.79** | **1.92** | **1.84** | **1.75** | **1.60** | **1.51** | **1.37** |
| 1GQ | 1.03 | 0.96 | 0.97 | 0.96 | 0.93 | 0.93 | 0.83 | 0.82 | 0.81 | 0.77 | 0.71 | 0.66 | 0.56 | 0.57 | 0.54 | 0.51 | 0.48 | 0.38 | 0.30 | 0.23 | 0.17 |
| 2GQ | 0.80 | 0.89 | 0.93 | 1.18 | 1.41 | 1.07 | 1.04 | 1.05 | 1.15 | 1.22 | 1.27 | 1.26 | 1.28 | 1.30 | 1.15 | 1.32 | 1.30 | 1.26 | 1.25 | 1.25 | 1.17 |
| 3GQ | **-** | **-** | **-** | **-** | **-** | 0.08 | 0.16 | 0.20 | 0.21 | 0.19 | 0.21 | 0.17 | 0.16 | 0.13 | 0.10 | 0.09 | 0.07 | 0.11 | 0.05 | 0.04 | 0.03 |
| **Germany** | **0.72** | **0.87** | **0.93** | **1.00** | **1.12** | **1.13** | **1.05** | **1.15** | **1.36** | **1.25** | **1.41** | **1.42** | **1.48** | **1.51** | **1.48** | **1.45** | **1.42** | **1.34** | **1.33** | **1.24** | **1.11** |
| 1GQ | 0.13 | 0.11 | 0.12 | 0.16 | 0.18 | 0.15 | 0.15 | 0.15 | 0.15 | 0.13 | 0.12 | 0.11 | 0.10 | 0.09 | 0.08 | 0.07 | 0.07 | 0.06 | 0.05 | 0.05 | 0.04 |
| 2GQ | 0.59 | 0.74 | 0.74 | 0.68 | 0.74 | 0.72 | 0.66 | 0.76 | 0.90 | 0.88 | 1.02 | 1.11 | 1.24 | 1.27 | 1.25 | 1.25 | 1.21 | 1.16 | 1.14 | 1.07 | 0.96 |
| 3GQ | **-** | 0.01 | 0.06 | 0.15 | 0.20 | 0.25 | 0.24 | 0.24 | 0.31 | 0.24 | 0.27 | 0.20 | 0.14 | 0.14 | 0.14 | 0.13 | 0.14 | 0.12 | 0.13 | 0.12 | 0.11 |
| **Greece** | **1.11** | **1.35** | **1.64** | **1.81** | **2.14** | **2.42** | **1.84** | ***1.83*** | ***1.84*** | ***2.12*** | ***2.93*** | ***2.97*** | **2.60** | ***2.85*** | **2.62** | **2.36** | **2.06** | **2.56** | **2.64** | **2.61** | **2.60** |
| 1GQ | 0.69 | 0.74 | 0.93 | 0.88 | 0.87 | 0.84 | 0.64 | *0.51* | *0.54* | *0.54* | *0.52* | *0.48* | 0.45 | *0.43* | 0.40 | 0.37 | 0.02 | 0.03 | 0.15 | 0.20 | 0.19 |
| 2GQ | 0.42 | 0.61 | 0.71 | 0.92 | 1.23 | 1.50 | 1.15 | *1.29* | *1.22* | *1.53* | *2.15* | *2.20* | 1.92 | *2.04* | 1.77 | 1.59 | 1.65 | 2.00 | 1.91 | 1.72 | 1.69 |
| 3GQ | **-** | **-** | **-** | 0.02 | 0.04 | 0.09 | 0.05 | *0.03* | *0.08* | *0.06* | *0.26* | *0.29* | 0.23 | *0.37* | 0.45 | 0.40 | 0.39 | 0.53 | 0.58 | 0.70 | 0.72 |
| **Hungary** | **-** | **0.87** | **1.09** | **1.03** | **1.20** | **1.35** | **1.52** | **1.65** | **1.91** | **1.80** | **1.51** | **1.76** | **1.79** | **1.97** | **1.94** | **1.97** | **2.11** | **2.43** | **2.71** | **2.38** | **2.40** |
| 1GQ | **-** | 0.31 | 0.35 | 0.34 | 0.38 | 0.46 | 0.49 | 0.48 | 0.52 | 0.52 | 0.42 | 0.41 | 0.40 | 0.40 | 0.35 | 0.33 | 0.33 | 0.33 | 0.32 | 0.30 | 0.27 |
| 2GQ | **-** | 0.56 | 0.74 | 0.69 | 0.81 | 0.84 | 0.96 | 1.06 | 1.22 | 1.14 | 1.01 | 1.29 | 1.34 | 1.50 | 1.52 | 1.58 | 1.73 | 2.00 | 2.25 | 1.96 | 2.00 |
| 3GQ | **-** | **-** | **-** | **-** | 0.01 | 0.05 | 0.07 | 0.11 | 0.17 | 0.14 | 0.08 | 0.06 | 0.06 | 0.07 | 0.08 | 0.06 | 0.05 | 0.10 | 0.14 | 0.12 | 0.13 |
| **Iceland** | ***0.43*** | ***0.55*** | ***0.62*** | ***0.63*** | ***0.71*** | ***0.71*** | ***0.71*** | ***0.64*** | ***0.79*** | **0.65** | **0.71** | **0.77** | **0.55** | ***1.02*** | ***1.05*** | ***1.01*** | ***1.12*** | **0.88** | **0.93** | **0.91** | **0.81** |
| 1GQ | *<0.01* | ***-*** | ***-*** | ***-*** | ***-*** | ***-*** | ***-*** | ***-*** | ***-*** | **-** | **-** | **-** | **-** | ***-*** | ***-*** | ***-*** | ***-*** | **-** | **-** | **-** | **-** |
| 2GQ | *0.43* | *0.55* | *0.62* | *0.63* | *0.71* | *0.71* | *0.71* | *0.64* | *0.79* | 0.65 | 0.71 | 0.77 | 0.55 | *1.01* | *1.05* | *1.01* | *1.12* | 0.88 | 0.93 | 0.91 | 0.81 |
| 3GQ | ***-*** | ***-*** | ***-*** | ***-*** | ***-*** | ***-*** | ***-*** | ***-*** | ***-*** | **-** | **-** | **-** | **-** | *<0.01* | *<0.01* | ***-*** | *<0.01* | <0.01 | <0.01 | <0.01 | <0.01 |
| **Ireland** | **-** | **0.52** | **0.56** | **0.57** | **0.64** | **0.65** | **0.72** | **0.75** | **0.84** | **0.94** | **1.04** | **1.04** | **0.94** | **0.91** | **0.93** | **0.87** | **0.87** | **0.84** | **0.92** | **0.87** | **0.81** |
| 1GQ | **-** | 0.10 | 0.09 | 0.08 | 0.08 | 0.08 | 0.04 | 0.01 | 0.01 | <0.01 | **-** | **-** | **-** | **-** | <0.01 | <0.01 | <0.01 | **-** | **-** | **-** | **-** |
| 2GQ | **-** | 0.42 | 0.47 | 0.45 | 0.51 | 0.54 | 0.64 | 0.70 | 0.80 | 0.88 | 0.96 | 0.96 | 0.88 | 0.85 | 0.88 | 0.82 | 0.82 | 0.80 | 0.87 | 0.83 | 0.77 |
| 3GQ | **-** | **-** | **-** | 0.03 | 0.05 | 0.04 | 0.04 | 0.04 | 0.04 | 0.06 | 0.08 | 0.08 | 0.06 | 0.05 | 0.05 | 0.05 | 0.05 | 0.04 | 0.05 | 0.04 | 0.04 |
| Country, community consumption of quinolones (J01M); 1GQ, consumption of first-generation quinolones; 2GQ, consumption of second-generation quinolones; 3GQ, consumption of third-generation  quinolones; **-**, no consumption reported; Numbers reported in *italic* are total care data, i.e. community and hospital sector combined; ^a^Data for Romania have a coverage in 2009 limited to 30-40%; ^b^Data for Spain include private prescriptions from 2016 onwards. | | | | | | | | | | | | | | | | | | | | | |
| **Italy** | **-** | **-** | **2.53** | **2.67** | **3.01** | **2.84** | **3.01** | **2.98** | **3.30** | **3.46** | **3.53** | **3.47** | **3.61** | **3.51** | **3.46** | **3.48** | **3.55** | **3.41** | **3.37** | **3.23** | **2.68** |
| 1GQ | **-** | **-** | 0.79 | 0.73 | 0.88 | 0.74 | 0.67 | 0.61 | 0.55 | 0.51 | 0.45 | 0.41 | 0.37 | 0.34 | 0.29 | 0.26 | 0.21 | 0.12 | 0.09 | 0.10 | 0.07 |
| 2GQ | **-** | **-** | 1.75 | 1.87 | 1.86 | 1.85 | 2.05 | 2.02 | 2.04 | 2.14 | 2.32 | 2.56 | 2.69 | 2.77 | 2.79 | 2.87 | 3.02 | 2.99 | 3.03 | 2.91 | 2.43 |
| 3GQ | **-** | **-** | **-** | 0.07 | 0.26 | 0.25 | 0.28 | 0.34 | 0.71 | 0.81 | 0.76 | 0.50 | 0.55 | 0.40 | 0.38 | 0.35 | 0.33 | 0.29 | 0.26 | 0.22 | 0.18 |
| **Latvia** | **-** | **-** | **-** | **-** | **-** | **0.88** | **-** | **0.92** | **1.05** | **1.13** | **1.09** | **1.02** | **0.89** | **0.91** | **0.98** | **1.03** | **1.05** | **1.05** | **1.05** | **1.05** | **1.03** |
| 1GQ | **-** | **-** | **-** | **-** | **-** | 0.31 | **-** | 0.25 | 0.29 | 0.27 | 0.28 | 0.27 | 0.23 | 0.23 | 0.23 | 0.22 | 0.22 | 0.22 | 0.21 | 0.19 | 0.19 |
| 2GQ | **-** | **-** | **-** | **-** | **-** | 0.58 | **-** | 0.66 | 0.76 | 0.86 | 0.81 | 0.75 | 0.65 | 0.67 | 0.75 | 0.81 | 0.84 | 0.83 | 0.85 | 0.86 | 0.85 |
| 3GQ | **-** | **-** | **-** | **-** | **-** | **-** | **-** | **-** | **-** | **-** | <0.01 | <0.01 | <0.01 | <0.01 | <0.01 | <0.01 | <0.01 | <0.01 | <0.01 | <0.01 | <0.01 |
| **Lithuania** | **-** | **-** | **-** | **-** | **-** | **-** | **-** | **-** | **-** | ***0.83*** | ***1.46*** | ***1.55*** | ***1.22*** | ***1.08*** | ***1.19*** | **1.00** | **0.96** | **0.91** | **0.91** | **0.88** | **0.87** |
| 1GQ | **-** | **-** | **-** | **-** | **-** | **-** | **-** | **-** | **-** | *0.32* | *0.47* | *0.48* | *0.34* | *0.29* | *0.29* | 0.25 | 0.23 | 0.21 | 0.19 | 0.16 | 0.17 |
| 2GQ | **-** | **-** | **-** | **-** | **-** | **-** | **-** | **-** | **-** | *0.52* | *0.99* | *1.06* | *0.87* | *0.78* | *0.88* | 0.75 | 0.73 | 0.70 | 0.72 | 0.72 | 0.69 |
| 3GQ | **-** | **-** | **-** | **-** | **-** | **-** | **-** | **-** | **-** | ***-*** | *<0.01* | *0.01* | *0.02* | *0.01* | *0.02* | <0.01 | <0.01 | <0.01 | <0.01 | <0.01 | <0.01 |
| **Luxembourg** | **1.63** | **1.52** | **1.86** | **2.29** | **2.61** | **2.48** | **2.81** | **2.49** | **2.66** | **2.59** | **2.80** | **2.77** | **2.80** | **2.85** | **2.84** | **2.77** | **2.65** | **2.57** | **2.48** | **2.41** | **2.77** |
| 1GQ | 0.60 | 0.56 | 0.53 | 0.47 | 0.40 | 0.34 | 0.32 | 0.31 | 0.29 | 0.28 | 0.26 | 0.27 | 0.26 | 0.25 | 0.25 | 0.23 | 0.15 | 0.10 | 0.11 | 0.09 | 0.12 |
| 2GQ | 1.03 | 0.96 | 1.33 | 1.82 | 2.21 | 1.93 | 2.14 | 1.85 | 1.92 | 1.87 | 2.02 | 2.03 | 2.04 | 2.07 | 2.06 | 2.02 | 2.00 | 2.04 | 1.93 | 1.87 | 2.19 |
| 3GQ | **-** | **-** | **-** | **-** | **-** | 0.21 | 0.36 | 0.33 | 0.44 | 0.45 | 0.52 | 0.47 | 0.51 | 0.53 | 0.54 | 0.52 | 0.50 | 0.43 | 0.44 | 0.44 | 0.45 |
| **Malta** | **-** | **-** | **-** | **-** | **-** | **-** | **-** | **-** | **-** | **-** | **1.70** | **1.78** | **1.65** | **1.78** | **1.86** | **2.01** | **2.92** | **3.06** | **2.64** | **2.37** | **2.18** |
| 1GQ | **-** | **-** | **-** | **-** | **-** | **-** | **-** | **-** | **-** | **-** | 0.34 | 0.33 | 0.31 | 0.28 | 0.16 | 0.22 | 0.20 | 0.23 | 0.19 | 0.13 | 0.19 |
| 2GQ | **-** | **-** | **-** | **-** | **-** | **-** | **-** | **-** | **-** | **-** | 1.20 | 1.29 | 1.18 | 1.39 | 1.59 | 1.67 | 2.66 | 2.80 | 2.43 | 2.23 | 1.98 |
| 3GQ | **-** | **-** | **-** | **-** | **-** | **-** | **-** | **-** | **-** | **-** | 0.16 | 0.16 | 0.16 | 0.11 | 0.10 | 0.11 | 0.07 | 0.03 | 0.02 | 0.01 | 0.01 |
| **Netherlands** | **0.84** | **0.84** | **0.89** | **0.85** | **0.83** | **0.81** | **0.81** | **0.84** | **0.86** | **0.91** | **0.93** | **0.91** | **0.89** | **0.87** | **0.83** | **0.81** | **0.76** | **0.79** | **0.77** | **0.75** | **0.73** |
| 1GQ | 0.43 | 0.41 | 0.40 | 0.37 | 0.35 | 0.32 | 0.31 | 0.31 | 0.29 | 0.30 | 0.28 | 0.26 | 0.24 | 0.21 | 0.18 | 0.16 | 0.12 | 0.09 | 0.07 | 0.06 | 0.04 |
| 2GQ | 0.40 | 0.43 | 0.49 | 0.47 | 0.49 | 0.48 | 0.49 | 0.51 | 0.53 | 0.56 | 0.59 | 0.61 | 0.61 | 0.62 | 0.62 | 0.62 | 0.61 | 0.68 | 0.67 | 0.66 | 0.67 |
| 3GQ | **-** | <0.01 | <0.01 | **-** | **-** | **-** | 0.01 | 0.02 | 0.04 | 0.05 | 0.06 | 0.03 | 0.04 | 0.04 | 0.03 | 0.03 | 0.03 | 0.03 | 0.03 | 0.02 | 0.03 |
| **Norway** | **-** | **0.25** | **-** | **-** | **0.35** | **0.40** | **0.40** | **0.43** | **0.48** | **0.44** | **0.48** | **0.50** | **0.51** | **0.54** | **0.55** | **0.56** | **0.54** | **0.50** | **0.46** | **0.41** | **0.35** |
| 1GQ | **-** | 0.01 | **-** | **-** | 0.01 | **-** | **-** | **-** | **-** | **-** | **-** | **-** | **-** | **-** | **-** | **-** | **-** | **-** | **-** | **-** | **-** |
| 2GQ | **-** | 0.24 | **-** | **-** | 0.34 | 0.40 | 0.40 | 0.43 | 0.48 | 0.44 | 0.48 | 0.50 | 0.51 | 0.54 | 0.55 | 0.55 | 0.53 | 0.50 | 0.45 | 0.40 | 0.34 |
| 3GQ | **-** | **-** | **-** | **-** | **-** | **-** | **-** | **-** | **-** | **-** | <0.01 | <0.01 | <0.01 | <0.01 | <0.01 | <0.01 | <0.01 | 0.01 | 0.01 | 0.01 | 0.01 |
| **Poland** | **-** | **1.24** | **1.12** | **0.97** | **1.04** | **1.11** | **-** | **1.00** | **1.14** | **-** | **1.15** | **1.21** | **1.25** | **1.23** | **1.23** | **1.19** | **1.18** | **1.21** | **1.40** | **1.42** | **1.49** |
| 1GQ | **-** | 0.67 | 0.59 | 0.56 | 0.54 | 0.52 | **-** | 0.42 | 0.51 | **-** | 0.47 | 0.49 | 0.49 | 0.48 | 0.44 | 0.36 | 0.31 | 0.30 | 0.31 | 0.30 | 0.30 |
| 2GQ | **-** | 0.56 | 0.53 | 0.42 | 0.50 | 0.59 | **-** | 0.59 | 0.63 | **-** | 0.68 | 0.72 | 0.76 | 0.76 | 0.79 | 0.82 | 0.87 | 0.90 | 1.02 | 1.04 | 1.10 |
| 3GQ | **-** | **-** | **-** | <0.01 | <0.01 | <0.01 | **-** | <0.01 | <0.01 | **-** | <0.01 | <0.01 | **-** | **-** | <0.01 | <0.01 | <0.01 | 0.01 | 0.07 | 0.08 | 0.10 |
| Country, community consumption of quinolones (J01M); 1GQ, consumption of first-generation quinolones; 2GQ, consumption of second-generation quinolones; 3GQ, consumption of third-generation  quinolones; **-**, no consumption reported; Numbers reported in *italic* are total care data, i.e. community and hospital sector combined; ^a^Data for Romania have a coverage in 2009 limited to 30-40%; ^b^Data for Spain include private prescriptions from 2016 onwards. | | | | | | | | | | | | | | | | | | | | | |
| **Portugal** | **3.04** | **2.98** | **3.09** | **3.20** | **3.65** | **3.70** | **3.10** | **3.05** | **3.04** | **2.92** | **-** | **3.07** | **3.04** | **2.98** | **2.69** | **2.47** | **2.18** | **2.12** | **2.05** | **1.92** | **1.26** |
| 1GQ | 0.56 | 0.59 | 0.63 | 0.66 | 0.65 | 0.58 | 0.42 | 0.40 | 0.33 | 0.30 | **-** | 0.24 | 0.23 | 0.21 | 0.23 | 0.22 | 0.15 | 0.15 | 0.14 | 0.13 | 0.12 |
| 2GQ | 2.48 | 2.39 | 2.46 | 2.54 | 2.91 | 2.82 | 2.29 | 2.27 | 2.35 | 2.35 | **-** | 2.23 | 2.24 | 2.26 | 2.03 | 1.87 | 1.72 | 1.65 | 1.63 | 1.54 | 1.05 |
| 3GQ | **-** | **-** | **-** | **-** | 0.08 | 0.31 | 0.38 | 0.37 | 0.36 | 0.27 | **-** | 0.59 | 0.56 | 0.50 | 0.43 | 0.38 | 0.31 | 0.32 | 0.28 | 0.25 | 0.09 |
| **Romania^a^** | **-** | **-** | **-** | **-** | **-** | **-** | **-** | **-** | **-** | **-** | **-** | **-** | ***1.25*** | **-** | ***3.39*** | ***3.36*** | ***3.47*** | ***3.72*** | ***3.50*** | ***2.89*** | ***3.25*** |
| 1GQ | **-** | **-** | **-** | **-** | **-** | **-** | **-** | **-** | **-** | **-** | **-** | **-** | *0.25* | **-** | *1.03* | *0.97* | *0.90* | *0.85* | *0.84* | *0.79* | *0.70* |
| 2GQ | **-** | **-** | **-** | **-** | **-** | **-** | **-** | **-** | **-** | **-** | **-** | **-** | *0.97* | **-** | *2.31* | *2.35* | *2.52* | *2.82* | *2.60* | *2.06* | *2.49* |
| 3GQ | **-** | **-** | **-** | **-** | **-** | **-** | **-** | **-** | **-** | **-** | **-** | **-** | *0.03* | **-** | *0.05* | *0.05* | *0.05* | *0.06* | *0.06* | *0.05* | *0.05* |
| **Slovakia** | **-** | **-** | **1.23** | **1.55** | **1.74** | **1.67** | **1.58** | **1.33** | **1.67** | **1.70** | **1.97** | **1.92** | **2.03** | **-** | ***2.41*** | **1.95** | **2.18** | **0.63** | **2.40** | **2.26** | **-** |
| 1GQ | **-** | **-** | 0.42 | 0.48 | 0.43 | 0.41 | 0.35 | 0.40 | 0.41 | 0.36 | 0.35 | 0.37 | 0.35 | **-** | *0.36* | 0.30 | 0.32 | 0.31 | 0.31 | 0.29 | **-** |
| 2GQ | **-** | **-** | 0.81 | 1.07 | 1.31 | 1.26 | 1.23 | 0.94 | 1.27 | 1.33 | 1.60 | 1.53 | 1.66 | **-** | *2.04* | 1.64 | 1.84 | 0.30 | 2.08 | 1.95 | **-** |
| 3GQ | **-** | **-** | **-** | **-** | **-** | **-** | **-** | **-** | **-** | <0.01 | 0.01 | 0.02 | 0.02 | **-** | *0.02* | 0.01 | 0.01 | 0.01 | 0.01 | 0.01 | **-** |
| **Slovenia** | **1.44** | **1.66** | **1.62** | **1.39** | **1.34** | **1.38** | **1.18** | **1.12** | **1.15** | **1.08** | **1.12** | **1.12** | **1.08** | **1.10** | **1.08** | **1.08** | **1.10** | **1.11** | **1.16** | **1.14** | **1.11** |
| 1GQ | 0.94 | 1.06 | 1.01 | 0.81 | 0.69 | 0.63 | 0.48 | 0.40 | 0.37 | 0.35 | 0.34 | 0.33 | 0.30 | 0.30 | 0.28 | 0.25 | 0.24 | 0.21 | 0.21 | 0.20 | 0.17 |
| 2GQ | 0.50 | 0.59 | 0.61 | 0.58 | 0.58 | 0.66 | 0.60 | 0.62 | 0.64 | 0.62 | 0.65 | 0.67 | 0.67 | 0.71 | 0.71 | 0.73 | 0.76 | 0.79 | 0.82 | 0.83 | 0.83 |
| 3GQ | **-** | **-** | **-** | <0.01 | 0.07 | 0.09 | 0.10 | 0.10 | 0.14 | 0.11 | 0.13 | 0.11 | 0.11 | 0.10 | 0.10 | 0.10 | 0.10 | 0.10 | 0.13 | 0.11 | 0.11 |
| **Spain^b^** | **2.18** | **2.12** | **2.17** | **2.18** | **2.22** | **2.20** | **2.24** | **2.25** | **2.26** | **2.32** | **2.47** | **2.43** | **2.42** | **2.54** | **2.57** | **2.46** | **2.36** | **2.31** | **2.35** | **2.90** | **2.82** |
| 1GQ | 0.96 | 0.86 | 0.79 | 0.77 | 0.70 | 0.61 | 0.58 | 0.52 | 0.47 | 0.44 | 0.39 | 0.36 | 0.33 | 0.32 | 0.29 | 0.25 | 0.24 | 0.22 | 0.20 | 0.23 | 0.20 |
| 2GQ | 1.23 | 1.26 | 1.33 | 1.27 | 1.31 | 1.30 | 1.32 | 1.38 | 1.40 | 1.52 | 1.64 | 1.72 | 1.76 | 1.89 | 1.96 | 1.92 | 1.87 | 1.87 | 1.93 | 2.45 | 2.43 |
| 3GQ | **-** | **-** | 0.05 | 0.14 | 0.21 | 0.29 | 0.34 | 0.35 | 0.38 | 0.36 | 0.44 | 0.35 | 0.33 | 0.33 | 0.32 | 0.29 | 0.26 | 0.23 | 0.22 | 0.22 | 0.19 |
| **Sweden** | **1.01** | **1.04** | **1.07** | **1.05** | **1.09** | **1.01** | **1.00** | **0.98** | **0.99** | **0.98** | **0.92** | **0.83** | **0.79** | **0.77** | **0.77** | **0.75** | **0.71** | **0.69** | **0.68** | **0.66** | **0.63** |
| 1GQ | 0.57 | 0.57 | 0.56 | 0.54 | 0.52 | 0.47 | 0.43 | 0.38 | 0.31 | 0.24 | 0.17 | 0.10 | 0.05 | 0.04 | 0.03 | 0.02 | 0.01 | 0.01 | 0.01 | 0.01 | 0.01 |
| 2GQ | 0.44 | 0.47 | 0.50 | 0.51 | 0.56 | 0.52 | 0.55 | 0.58 | 0.66 | 0.72 | 0.73 | 0.72 | 0.72 | 0.73 | 0.73 | 0.72 | 0.69 | 0.67 | 0.66 | 0.64 | 0.61 |
| 3GQ | **-** | <0.01 | <0.01 | 0.01 | 0.01 | 0.02 | 0.03 | 0.02 | 0.02 | 0.02 | 0.02 | 0.01 | 0.01 | 0.01 | 0.01 | 0.01 | 0.01 | 0.01 | 0.01 | 0.01 | 0.01 |
| **United Kingdom** | **0.48** | **0.47** | **0.43** | **0.42** | **0.44** | **0.45** | **0.45** | **0.48** | **0.52** | **0.53** | **0.62** | **0.53** | **0.48** | **0.46** | **0.43** | **0.42** | **0.49** | **0.48** | **0.46** | **0.44** | **0.45** |
| 1GQ | 0.06 | 0.05 | 0.05 | 0.04 | 0.04 | 0.04 | 0.03 | 0.03 | 0.03 | 0.03 | 0.08 | 0.02 | 0.02 | 0.02 | 0.01 | 0.01 | 0.01 | <0.01 | <0.01 | **-** | **-** |
| 2GQ | 0.42 | 0.42 | 0.39 | 0.38 | 0.40 | 0.41 | 0.42 | 0.44 | 0.49 | 0.50 | 0.53 | 0.49 | 0.46 | 0.44 | 0.41 | 0.40 | 0.47 | 0.47 | 0.45 | 0.43 | 0.44 |
| 3GQ | **-** | **-** | **-** | **-** | **-** | **-** | <0.01 | <0.01 | <0.01 | 0.01 | 0.01 | 0.01 | 0.01 | 0.01 | 0.01 | 0.01 | 0.01 | 0.01 | 0.01 | 0.01 | 0.01 |

Country, community consumption of quinolones (J01M); 1GQ, consumption of first-generation quinolones; 2GQ, consumption of second-generation quinolones; 3GQ, consumption of third-generation quinolones; **-**, no consumption reported; Numbers reported in *italic* are total care data, i.e. community and hospital sector combined; ^a^Data for Romania have a coverage in 2009 limited to 30-40%; ^b^Data for Spain include
private prescriptions from 2016 onwards.

**Table S2. Consumption of quinolones (ATC J01M) in the community, expressed in packages per 1000 inhabitants per day, 23 EU/EEA countries, 2006−2017.**

| **Country** | **2006** | **2007** | **2008** | **2009** | **2010** | **2011** | **2012** | **2013** | **2014** | **2015** | **2016** | **2017** |  |
| --- | --- | --- | --- | --- | --- | --- | --- | --- | --- | --- | --- | --- | --- |
| **Austria** | **-** | **0.23** | **0.21** | **0.22** | **0.22** | **0.21** | **0.21** | **0.23** | **0.20** | **0.20** | **0.19** | **0.19** |  |
| 1GQ | **-** | 0.03 | 0.03 | 0.02 | 0.02 | 0.02 | 0.02 | 0.02 | 0.01 | 0.01 | 0.01 | <0.01 |  |
| 2GQ | **-** | 0.16 | 0.15 | 0.16 | 0.15 | 0.15 | 0.15 | 0.17 | 0.15 | 0.14 | 0.14 | 0.14 |  |
| 3GQ | **-** | 0.05 | 0.03 | 0.04 | 0.04 | 0.04 | 0.04 | 0.05 | 0.04 | 0.05 | 0.04 | 0.05 |  |
| **Belgium^a^** | **-** | **0.28** | **0.29** | **0.31** | **0.32** | **0.32** | **0.32** | **0.31** | **0.29** | **0.29** | **0.36** | **0.31** |  |
| 1GQ | **-** | 0.04 | 0.04 | 0.04 | 0.03 | 0.03 | 0.03 | 0.02 | 0.02 | 0.02 | 0.02 | 0.01 |  |
| 2GQ | **-** | 0.17 | 0.19 | 0.18 | 0.18 | 0.18 | 0.17 | 0.17 | 0.17 | 0.16 | 0.22 | 0.20 |  |
| 3GQ | **-** | 0.07 | 0.07 | 0.09 | 0.10 | 0.11 | 0.12 | 0.11 | 0.11 | 0.11 | 0.12 | 0.10 |  |
| **Bulgaria** | **0.33** | **0.36** | **0.38** | **0.36** | **0.36** | **0.40** | **0.42** | **0.44** | **0.50** | **0.49** | **0.47** | **0.49** |  |
| 1GQ | 0.03 | 0.03 | 0.04 | 0.03 | 0.03 | 0.03 | 0.02 | 0.02 | 0.02 | 0.02 | 0.03 | 0.02 |  |
| 2GQ | 0.30 | 0.32 | 0.34 | 0.31 | 0.31 | 0.36 | 0.39 | 0.41 | 0.46 | 0.45 | 0.42 | 0.43 |  |
| 3GQ | <0.01 | 0.01 | 0.01 | 0.01 | 0.01 | 0.01 | 0.01 | 0.01 | 0.01 | 0.02 | 0.03 | 0.03 |  |
| **Croatia** | **-** | **0.20** | **0.21** | **0.20** | **0.19** | **0.18** | **0.20** | **0.19** | **0.20** | **0.20** | **0.21** | **0.21** |  |
| 1GQ | **-** | 0.10 | 0.09 | 0.08 | 0.08 | 0.08 | 0.09 | 0.09 | 0.08 | 0.08 | 0.08 | 0.07 |  |
| 2GQ | **-** | 0.09 | 0.10 | 0.11 | 0.10 | 0.08 | 0.10 | 0.10 | 0.11 | 0.12 | 0.12 | 0.13 |  |
| 3GQ | **-** | 0.02 | 0.02 | 0.02 | 0.02 | 0.01 | 0.01 | 0.01 | 0.01 | 0.01 | 0.01 | 0.01 |  |
| **Czechia** | **-** | **0.19** | **-** | **-** | **0.33** | **0.31** | **0.27** | **0.24** | **0.25** | **0.24** | **-** | **-** |  |
| 1GQ | **-** | 0.06 | **-** | **-** | 0.06 | 0.05 | 0.05 | 0.02 | 0.02 | 0.01 | **-** | **-** |  |
| 2GQ | **-** | 0.13 | **-** | **-** | 0.27 | 0.26 | 0.22 | 0.22 | 0.23 | 0.23 | **-** | **-** |  |
| 3GQ | **-** | **-** | **-** | **-** | **-** | <0.01 | <0.01 | <0.01 | <0.01 | <0.01 | **-** | **-** |  |
| **Denmark** | **-** | **0.06** | **0.07** | **0.07** | **0.07** | **0.07** | **0.07** | **0.07** | **0.06** | **0.06** | **0.06** | **0.06** |  |
| 1GQ | **-** | **-** | **-** | **-** | **-** | **-** | **-** | **-** | **-** | **-** | **-** | **-** |  |
| 2GQ | **-** | 0.06 | 0.07 | 0.07 | 0.06 | 0.07 | 0.07 | 0.06 | 0.06 | 0.06 | 0.05 | 0.05 |  |
| 3GQ | **-** | <0.01 | <0.01 | <0.01 | <0.01 | <0.01 | <0.01 | <0.01 | <0.01 | <0.01 | 0.01 | 0.01 |  |
| **Estonia** | **0.15** | **0.16** | **0.16** | **0.14** | **0.15** | **0.15** | **0.16** | **0.16** | **0.16** | **0.17** | **0.16** | **0.14** |  |
| 1GQ | 0.03 | 0.03 | 0.03 | 0.03 | 0.03 | 0.03 | 0.03 | 0.03 | 0.02 | 0.02 | 0.02 | 0.02 |  |
| 2GQ | 0.12 | 0.13 | 0.13 | 0.11 | 0.12 | 0.13 | 0.13 | 0.14 | 0.14 | 0.15 | 0.14 | 0.13 |  |
| 3GQ | **-** | **-** | <0.01 | <0.01 | <0.01 | <0.01 | <0.01 | <0.01 | <0.01 | <0.01 | <0.01 | <0.01 |  |
| Country, community consumption of quinolones (J01M); 1GQ, consumption of first-generation quinolones; 2GQ, consumption of second-generation quinolones; 3GQ, consumption of third-generation quinolones; **-**, no consumption reported; Numbers reported in *italic* are total care data, i.e. community and hospital sector combined; ^a^Data for Belgium are slightly overestimated from 2016 onwards (nursing homes counting units versus packages before 2016); ^b^Data for the Netherlands are based on average package size; ^c^Data for Spain include private prescriptions from 2016 onwards. | | | | | | | | | | | | | |
| **Finland** | **-** | **-** | **0.11** | **0.12** | **0.12** | **0.13** | **0.12** | **0.11** | **0.10** | **0.09** | **0.09** | **0.08** |  |
| 1GQ | **-** | **-** | 0.02 | 0.02 | 0.02 | 0.01 | 0.01 | 0.01 | 0.01 | <0.01 | **-** | **-** |  |
| 2GQ | **-** | **-** | 0.09 | 0.09 | 0.10 | 0.10 | 0.10 | 0.09 | 0.09 | 0.09 | 0.08 | 0.07 |  |
| 3GQ | **-** | **-** | 0.01 | 0.01 | 0.01 | 0.01 | 0.01 | 0.01 | 0.01 | 0.01 | 0.01 | 0.01 |  |
| **France** | **-** | **-** | **-** | **-** | **0.41** | **0.36** | **0.39** | **0.37** | **0.35** | **0.32** | **0.30** | **0.27** |  |
| 1GQ | **-** | **-** | **-** | **-** | 0.11 | 0.11 | 0.10 | 0.09 | 0.08 | 0.06 | 0.05 | 0.03 |  |
| 2GQ | **-** | **-** | **-** | **-** | 0.27 | 0.24 | 0.27 | 0.27 | 0.26 | 0.25 | 0.25 | 0.24 |  |
| 3GQ | **-** | **-** | **-** | **-** | 0.02 | 0.02 | 0.01 | 0.01 | 0.02 | 0.01 | 0.01 | 0.01 |  |
| **Greece** | ***0.65*** | ***0.89*** | ***0.91*** | **0.57** | ***0.85*** | **0.55** | **0.47** | **0.44** | **0.53** | **0.54** | **0.50** | **0.55** |  |
| 1GQ | *0.08* | *0.07* | *0.07* | 0.06 | *0.06* | 0.06 | 0.05 | <0.01 | <0.01 | 0.02 | 0.03 | 0.03 |  |
| 2GQ | *0.55* | *0.75* | *0.77* | 0.46 | *0.70* | 0.41 | 0.35 | 0.37 | 0.43 | 0.42 | 0.35 | 0.39 |  |
| 3GQ | *0.02* | *0.07* | *0.07* | 0.04 | *0.09* | 0.08 | 0.07 | 0.07 | 0.09 | 0.10 | 0.12 | 0.13 |  |
| **Iceland** | **-** | **-** | **-** | **-** | ***0.14*** | ***0.16*** | ***0.15*** | ***0.17*** | **0.11** | **0.11** | **0.11** | **0.10** |  |
| 1GQ | **-** | **-** | **-** | **-** | ***-*** | ***-*** | ***-*** | ***-*** | **-** | **-** | **-** | **-** |  |
| 2GQ | **-** | **-** | **-** | **-** | *0.14* | *0.16* | *0.15* | *0.17* | 0.11 | 0.11 | 0.11 | 0.10 |  |
| 3GQ | **-** | **-** | **-** | **-** | *<0.01* | *<0.01* | ***-*** | *<0.01* | <0.01 | **-** | **-** | **-** |  |
| **Ireland** | **-** | **0.16** | **-** | **-** | **0.14** | **0.14** | **0.13** | **0.13** | **0.12** | **0.13** | **0.13** | **0.12** |  |
| 1GQ | **-** |  | **-** | **-** | **-** | **-** | **-** | **-** | **-** | **-** | **-** | **-** |  |
| 2GQ | **-** | 0.14 | **-** | **-** | 0.13 | 0.13 | 0.12 | 0.12 | 0.12 | 0.12 | 0.12 | 0.11 |  |
| 3GQ | **-** | 0.02 | **-** | **-** | 0.01 | 0.01 | 0.01 | 0.01 | 0.01 | 0.01 | 0.01 | 0.01 |  |
| **Italy** | **-** | **-** | **0.80** | **-** | **0.81** | **0.80** | **0.81** | **0.82** | **0.80** | **0.79** | **0.74** | **0.64** |  |
| 1GQ | **-** | **-** | 0.05 | **-** | 0.04 | 0.04 | 0.03 | 0.03 | 0.02 | 0.01 | 0.01 | 0.01 |  |
| 2GQ | **-** | **-** | 0.65 | **-** | 0.69 | 0.69 | 0.70 | 0.73 | 0.73 | 0.73 | 0.68 | 0.60 |  |
| 3GQ | **-** | **-** | 0.10 | **-** | 0.08 | 0.08 | 0.07 | 0.07 | 0.06 | 0.05 | 0.05 | 0.04 |  |
| **Latvia** | **-** | **-** | **-** | **-** | **0.18** | **0.19** | **0.20** | **0.20** | **0.20** | **0.19** | **0.19** | **0.19** |  |
| 1GQ | **-** | **-** | **-** | **-** | 0.04 | 0.04 | 0.03 | 0.03 | 0.03 | 0.02 | 0.02 | 0.02 |  |
| 2GQ | **-** | **-** | **-** | **-** | 0.14 | 0.15 | 0.16 | 0.17 | 0.17 | 0.17 | 0.17 | 0.17 |  |
| 3GQ | **-** | **-** | **-** | **-** | <0.01 | <0.01 | <0.01 | <0.01 | <0.01 | <0.01 | <0.01 | <0.01 |  |
| **Lithuania** | **-** | ***0.30*** | ***0.33*** | ***0.26*** | ***0.24*** | ***0.26*** | **0.19** | **0.19** | **0.17** | **0.16** | **0.16** | **0.16** |  |
| 1GQ | **-** | *0.09* | *0.08* | *0.06* | *0.05* | *0.05* | 0.04 | 0.04 | 0.03 | 0.02 | 0.02 | 0.02 |  |
| 2GQ | **-** | *0.22* | *0.24* | *0.20* | *0.19* | *0.21* | 0.15 | 0.15 | 0.14 | 0.14 | 0.14 | 0.13 |  |
| 3GQ | **-** | *<0.01* | *<0.01* | *<0.01* | *<0.01* | *<0.01* | <0.01 | <0.01 | <0.01 | <0.01 | <0.01 | <0.01 |  |
| Country, community consumption of quinolones (J01M); 1GQ, consumption of first-generation quinolones; 2GQ, consumption of second-generation quinolones; 3GQ, consumption of third-generation quinolones; **-**, no consumption reported; Numbers reported in *italic* are total care data, i.e. community and hospital sector combined; ^a^Data for Belgium are slightly overestimated from 2016 onwards (nursing homes counting units versus packages before 2016); ^b^Data for the Netherlands are based on average package size; ^c^Data for Spain include private prescriptions from 2016 onwards. | | | | | | | | | | | | | |
| **Luxembourg** | **0.38** | **0.40** | **0.39** | **0.39** | **0.38** | **0.37** | **0.36** | **0.34** | **0.32** | **0.31** | **0.30** | **-** |  |
| 1GQ | 0.06 | 0.06 | 0.06 | 0.05 | 0.05 | 0.04 | 0.04 | 0.02 | 0.01 | 0.01 | 0.01 | **-** |  |
| 2GQ | 0.27 | 0.28 | 0.27 | 0.27 | 0.27 | 0.26 | 0.25 | 0.25 | 0.26 | 0.24 | 0.23 | **-** |  |
| 3GQ | 0.06 | 0.07 | 0.06 | 0.06 | 0.07 | 0.06 | 0.06 | 0.06 | 0.05 | 0.05 | 0.05 | **-** |  |
| **Netherlands^b^** | **-** | **-** | **0.12** | **0.11** | **-** | **-** | **-** | **-** | **-** | **-** | **-** | **-** |  |
| 1GQ | **-** | **-** | 0.04 | 0.03 | **-** | **-** | **-** | **-** | **-** | **-** | **-** | **-** |  |
| 2GQ | **-** | **-** | 0.07 | 0.07 | **-** | **-** | **-** | **-** | **-** | **-** | **-** | **-** |  |
| 3GQ | **-** | **-** | 0.01 | 0.01 | **-** | **-** | **-** | **-** | **-** | **-** | **-** | **-** |  |
| **Portugal** | **-** | **-** | **0.38** | **0.37** | **0.36** | **0.33** | **0.30** | **0.26** | **0.26** | **0.25** | **0.23** | **0.21** |  |
| 1GQ | **-** | **-** | 0.03 | 0.03 | 0.03 | 0.03 | 0.03 | 0.02 | 0.02 | 0.02 | 0.02 | 0.02 |  |
| 2GQ | **-** | **-** | 0.27 | 0.27 | 0.28 | 0.25 | 0.23 | 0.21 | 0.20 | 0.20 | 0.19 | 0.17 |  |
| 3GQ | **-** | **-** | 0.07 | 0.07 | 0.06 | 0.05 | 0.05 | 0.04 | 0.04 | 0.03 | 0.03 | 0.03 |  |
| **Slovakia** | **-** | **-** | **-** | **-** | **-** | ***0.61*** | **0.36** | **0.41** | **0.43** | **0.46** | **0.43** | **-** |  |
| 1GQ | **-** | **-** | **-** | **-** | **-** | *0.04* | 0.03 | 0.03 | 0.03 | 0.03 | 0.03 | **-** |  |
| 2GQ | **-** | **-** | **-** | **-** | **-** | *0.58* | 0.32 | 0.38 | 0.39 | 0.42 | 0.40 | **-** |  |
| 3GQ | **-** | **-** | **-** | **-** | **-** | *<0.01* | <0.01 | <0.01 | 0.01 | <0.01 | <0.01 | **-** |  |
| **Slovenia** | **-** | **0.19** | **0.19** | **0.18** | **0.19** | **0.18** | **0.18** | **0.19** | **0.19** | **0.20** | **0.19** | **0.19** |  |
| 1GQ | **-** | 0.04 | 0.03 | 0.03 | 0.03 | 0.03 | 0.03 | 0.02 | 0.02 | 0.02 | 0.02 | 0.02 |  |
| 2GQ | **-** | 0.13 | 0.14 | 0.13 | 0.14 | 0.14 | 0.14 | 0.15 | 0.15 | 0.15 | 0.16 | 0.15 |  |
| 3GQ | **-** | 0.02 | 0.02 | 0.02 | 0.02 | 0.02 | 0.02 | 0.02 | 0.02 | 0.02 | 0.02 | 0.02 |  |
| **Spain^c^** | **-** | **-** | **-** | **-** | **0.31** | **0.31** | **0.29** | **0.29** | **0.30** | **0.31** | **0.38** | **0.37** |  |
| 1GQ | **-** | **-** | **-** | **-** | 0.04 | 0.04 | 0.04 | 0.03 | 0.03 | 0.03 | 0.03 | 0.03 |  |
| 2GQ | **-** | **-** | **-** | **-** | 0.21 | 0.22 | 0.21 | 0.22 | 0.23 | 0.24 | 0.31 | 0.31 |  |
| 3GQ | **-** | **-** | **-** | **-** | 0.05 | 0.05 | 0.05 | 0.04 | 0.04 | 0.04 | 0.04 | 0.03 |  |
| **Sweden** | **-** |  | **-** | **0.09** | **0.09** | **0.09** | **0.08** | **0.08** | **0.08** | **0.07** | **0.07** | **0.07** |  |
| 1GQ | **-** |  | **-** | 0.01 | 0.01 | <0.01 | <0.01 | <0.01 | <0.01 | <0.01 | <0.01 | <0.01 |  |
| 2GQ | **-** | **-** | **-** | 0.08 | 0.08 | 0.08 | 0.08 | 0.08 | 0.07 | 0.07 | 0.07 | 0.07 |  |
| 3GQ | **-** | **-** | **-** | <0.01 | <0.01 | <0.01 | <0.01 | <0.01 | <0.01 | <0.01 | <0.01 | <0.01 |  |
| **United Kingdom** | **-** | **-** | **-** | **-** | **-** | **-** | **-** | **-** | **-** | **<0.01** | **-** | **-** |  |
| 1GQ | **-** | **-** | **-** | **-** | **-** | **-** | **-** | **-** | **-** | **-** | **-** | **-** |  |
| 2GQ | **-** | **-** | **-** | **-** | **-** | **-** | **-** | **-** | **-** | <0.01 | **-** | **-** |  |
| 3GQ | **-** | **-** | **-** | **-** | **-** | **-** | **-** | **-** | **-** | **-** | **-** | **-** |  |

Country, community consumption of quinolones (J01M); 1GQ, consumption of first-generation quinolones; 2GQ, consumption of second-generation quinolones; 3GQ, consumption of third-generation
quinolones; **-**, no consumption reported; Numbers reported in *italic* are total care data, i.e. community and hospital sector combined; ^a^Data for Belgium are slightly overestimated from 2016 onwards (nursing homes counting units versus packages before 2016); ^b^Data for the Netherlands are based on average package size; ^c^Data for Spain include private prescriptions from 2016 onwards.

**
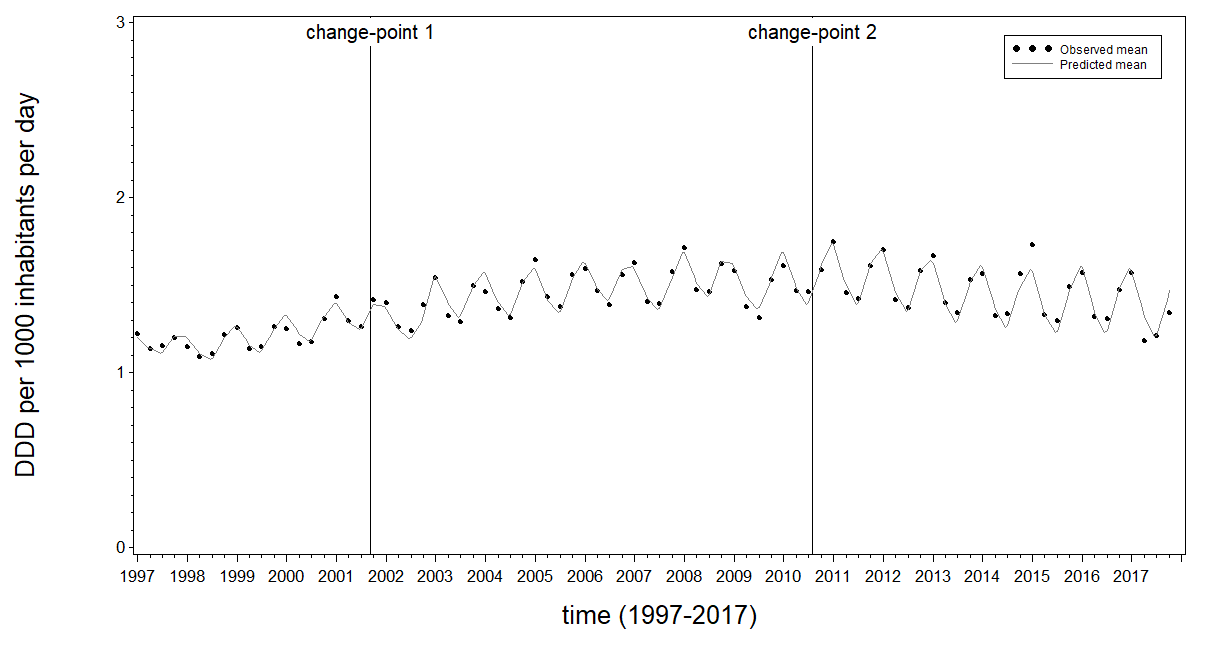
Figure S1. Average of observed (dots) and predicted (solid line) consumption of quinolones (ATC J01M) in the community expressed in DDD (ATC/DDD index 2019) per 1000 inhabitants per day and based on quarterly data, 25 EU/EEA countries, 1997-2017.**


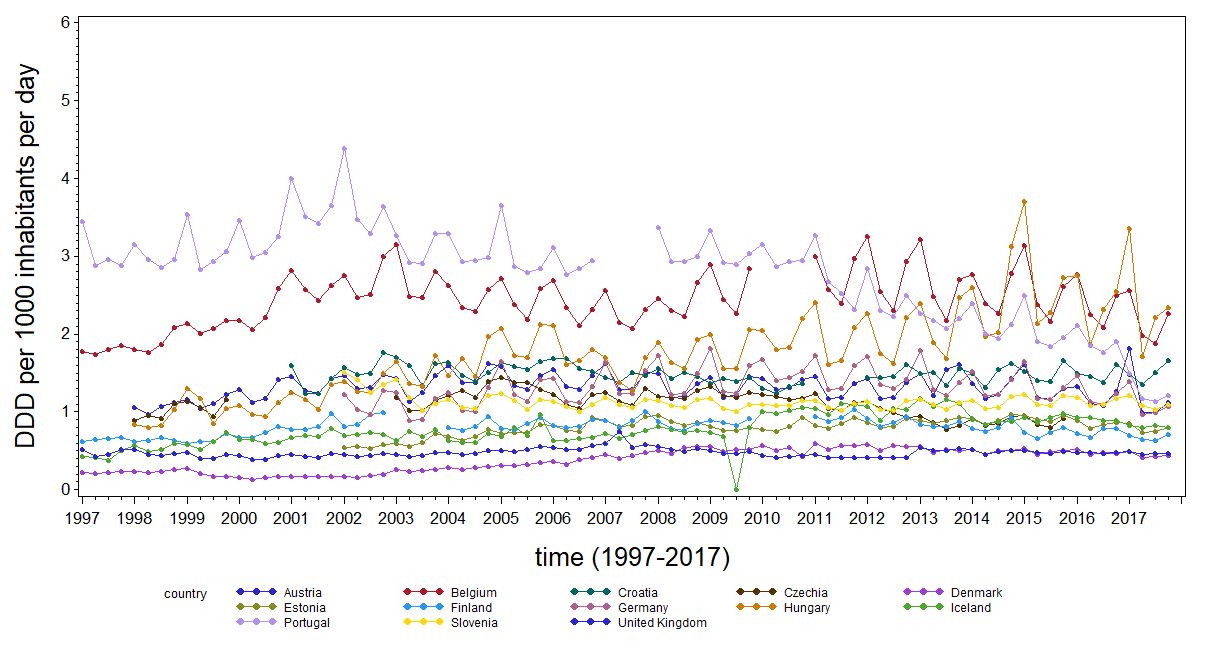


**Figure S2. Seasonal variation in consumption of quinolones (ATC J01M) in the community, expressed in DDD (ATC/DDD index 2019) per 1000 inhabitants per day, 13 EU/EEA countries reporting consumption per quarter for at least 15 years, 1997-2017.**


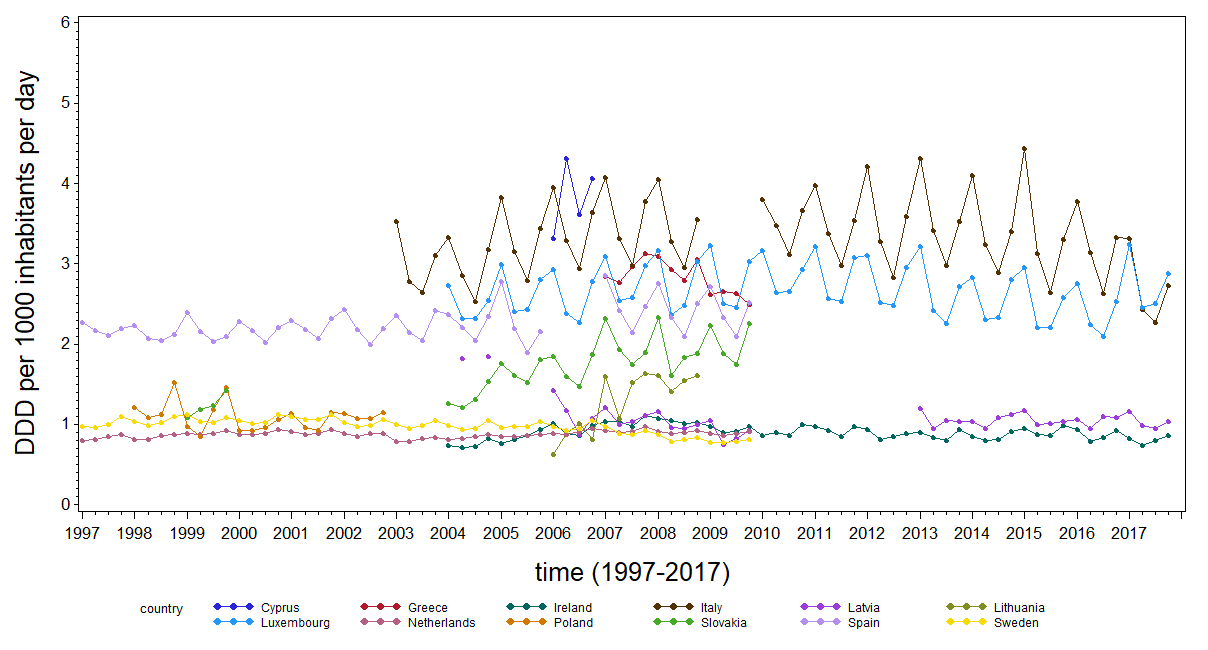


**Figure S3. Seasonal variation in consumption of quinolones (ATC J01M) in the community, expressed in DDD (ATC/DDD index 2019) per 1000 inhabitants per day, 12 EU countries reporting consumption per quarter for less than 15 years, 1997-2017. For Cyprus, total care data, i.e. community and hospital sector combined, are used. For Spain, private prescriptions are included from 2016 onwards.**

^
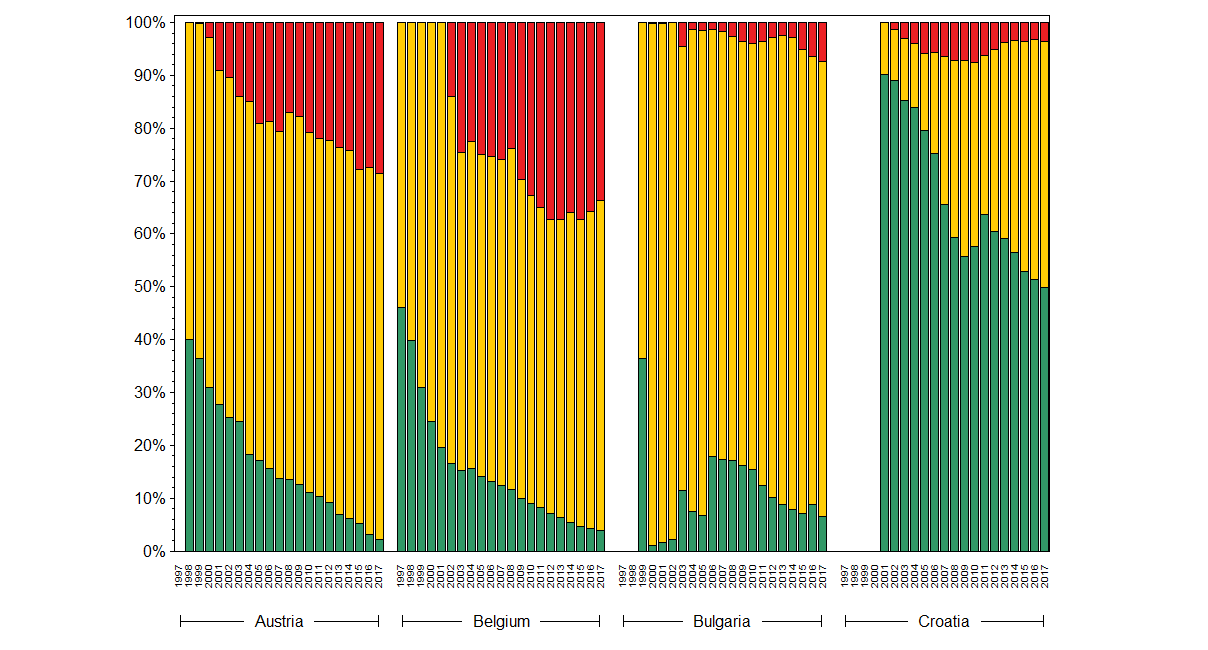
^

^
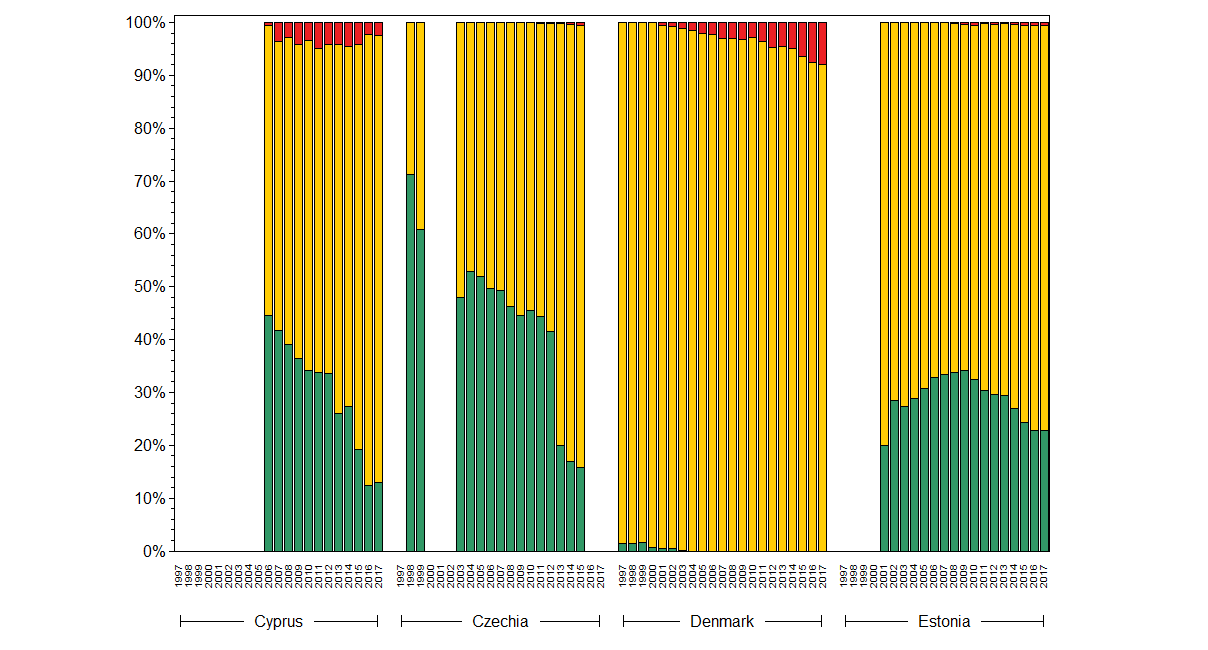
^

= first-generation quinolones, = second-generation quinolones, = third-generation quinolones
**Figure S4. Composition of quinolone (ATC J01M) consumption in the community, expressed in DDD (ATC/DDD index 2019) per 1000 inhabitants per day, 30 EU/EEA countries, 1997-2017. For Cyprus and Romania, total care data, i.e. community and hospital sector combined, are used. For Spain, private prescription are included from 2016 onwards. For Romania, data have a coverage in 2009 limited to 30-40%.**

^
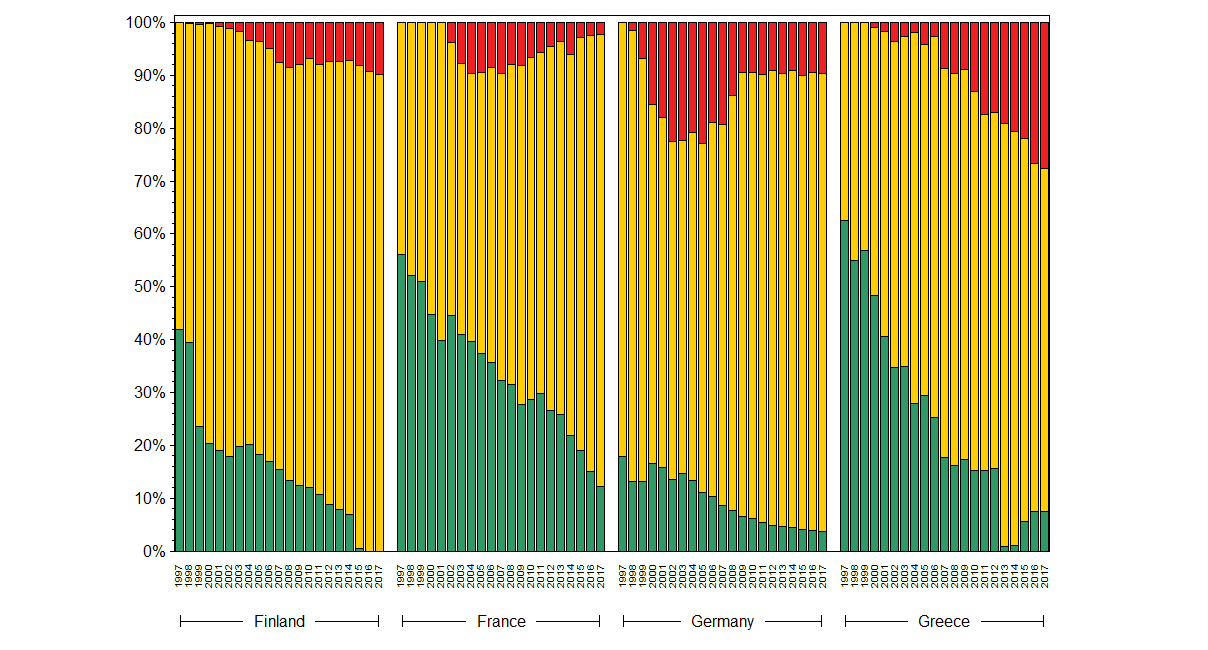

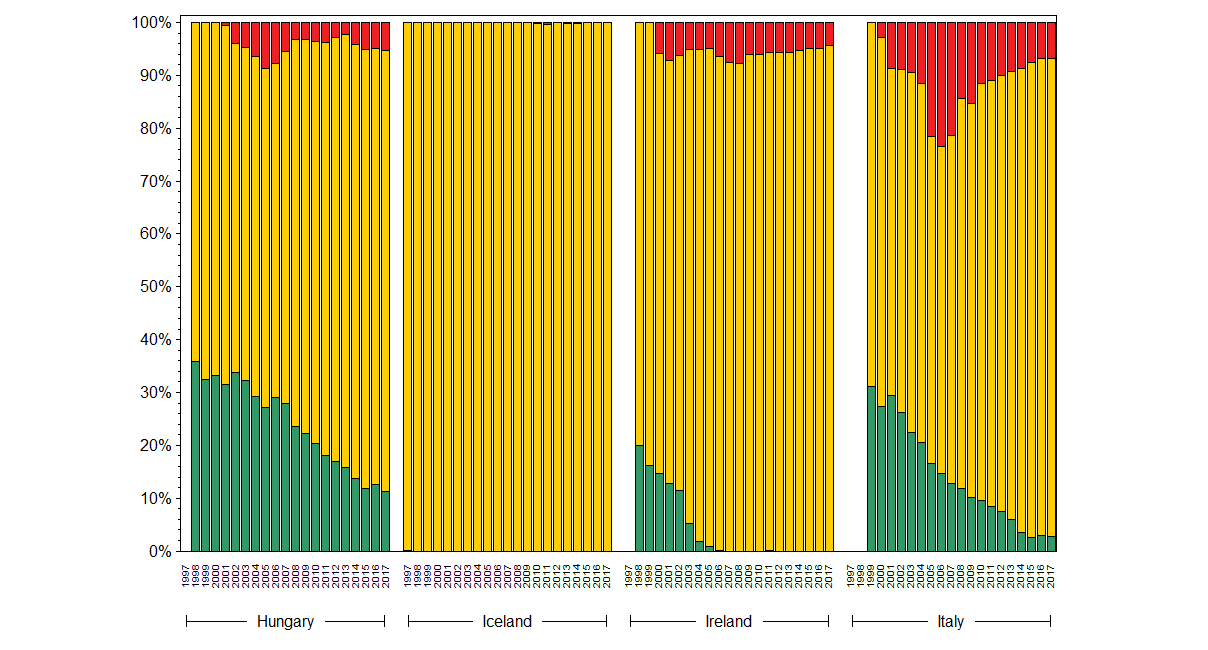
^

= first-generation quinolones, = second-generation quinolones, = third-generation quinolones
**Figure S4.** *Continued*

^
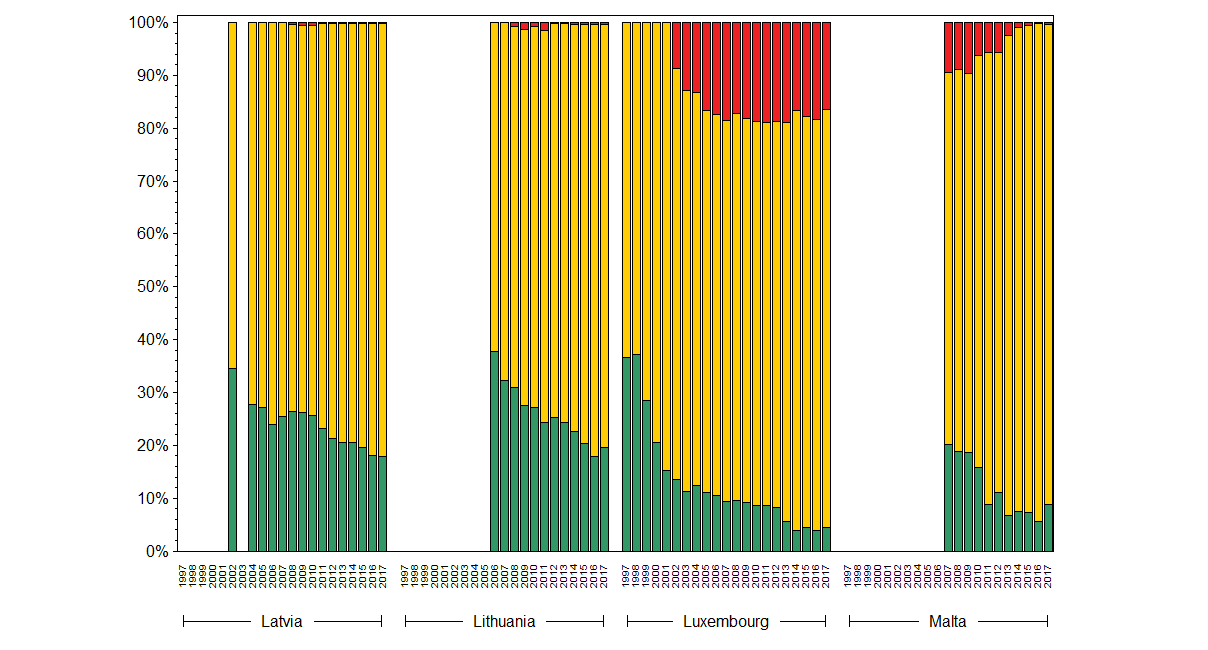

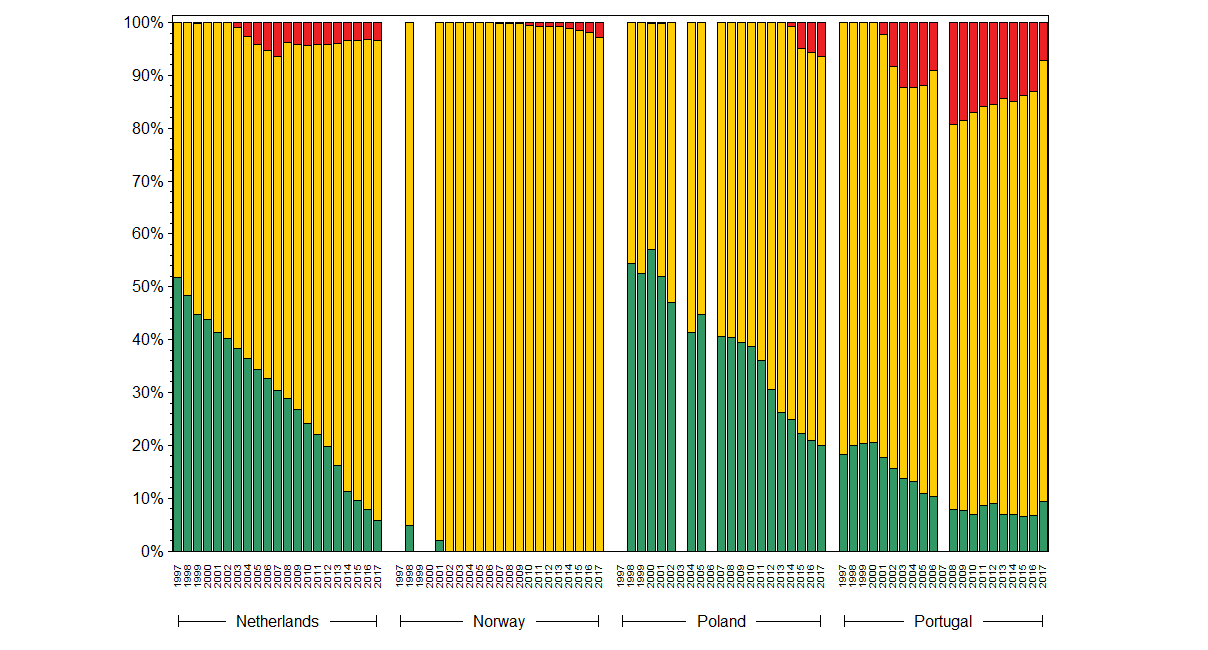
^

= first-generation quinolones, = second-generation quinolones, = third-generation quinolones
**Figure S4.** *Continued*

^
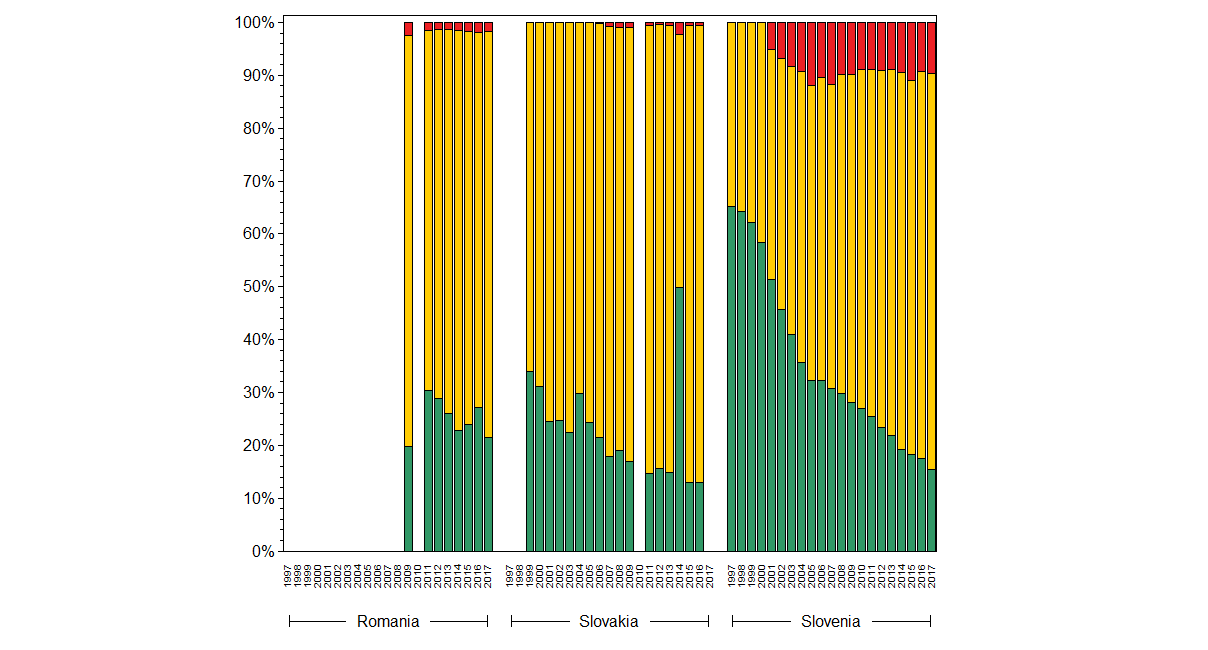
^


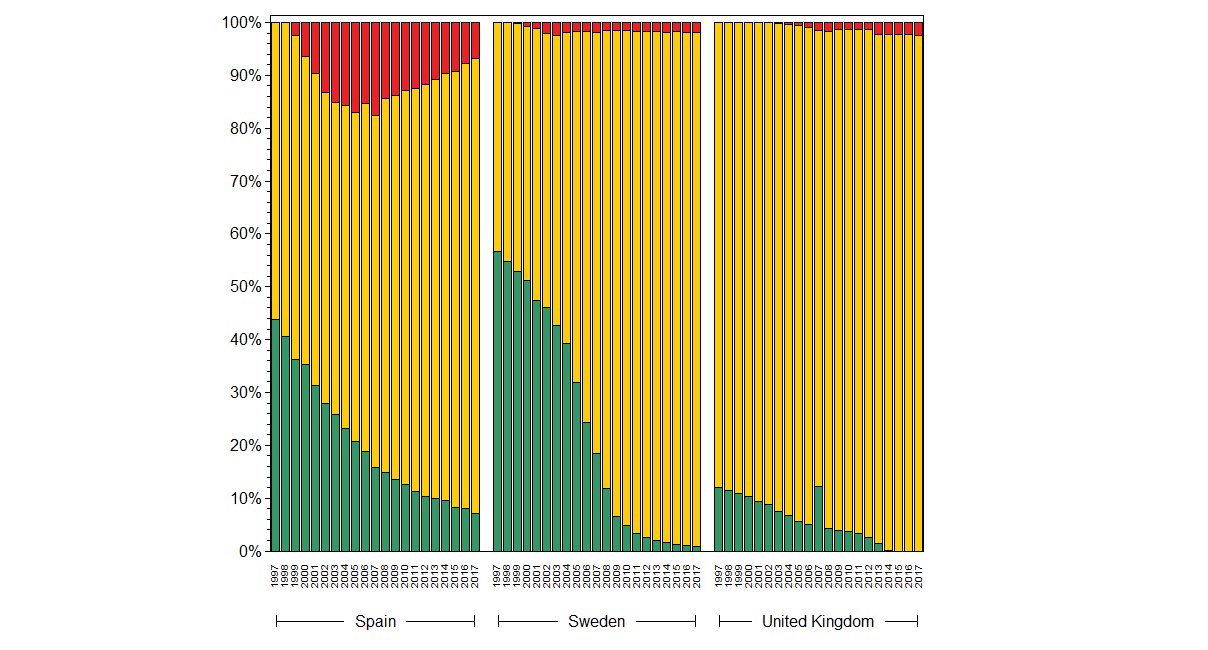


= first-generation quinolones, = second-generation quinolones, = third-generation quinolones
**Figure S4.** *Continued*

**
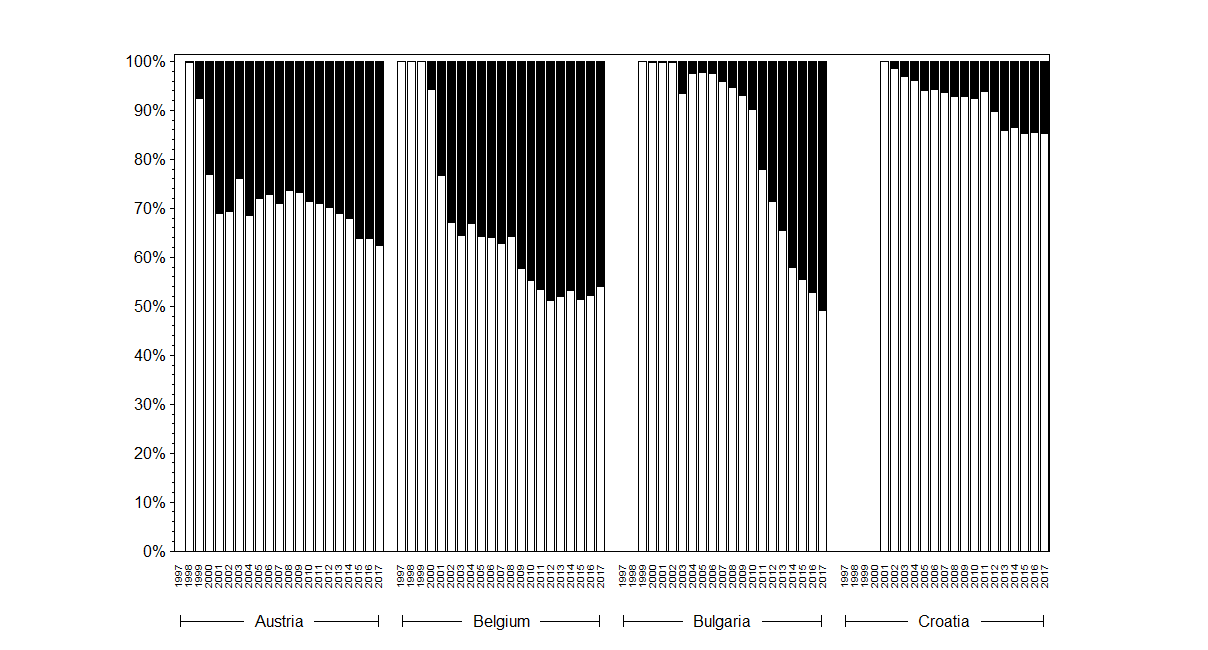

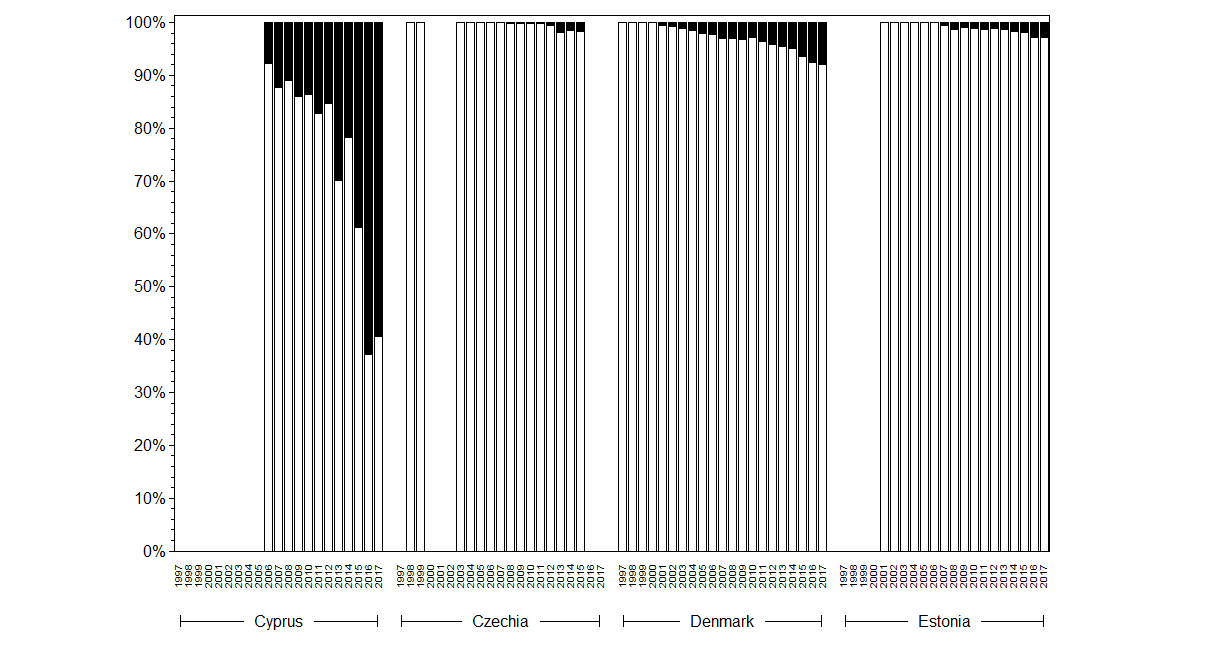
**

= levofloxacin and moxifloxacin, = other quinolones

**Figure S5. Composition of quinolone consumption (ATC J01M) in the community, levofloxacin (J01MA12) and moxifloxacin (J01MA14) *versus* other quinolones, expressed in DDD (ATC/DDD index 2019) per 1000 inhabitants per day, 30 EU/EEA countries, 1997-2017. For Cyprus and Romania, total care data, i.e. community and hospital sector combined, are used. For Spain, private prescription are included from 2016 onwards. For Romania, data have a coverage in 2009 limited to 30-40%.**

**
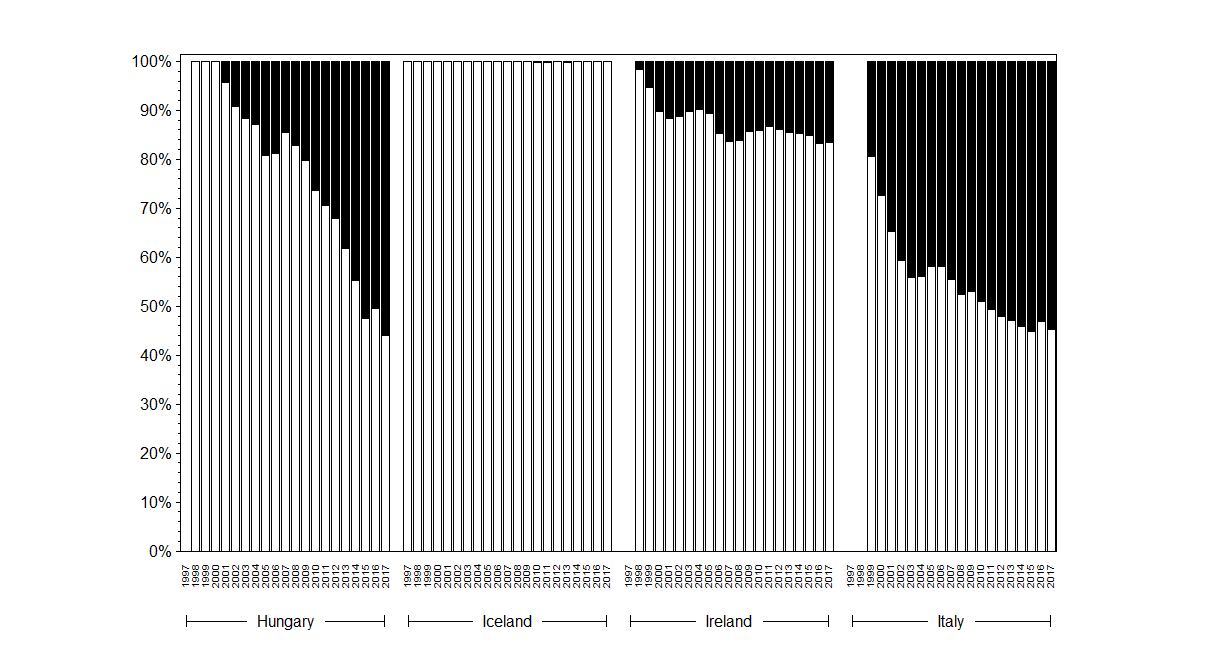

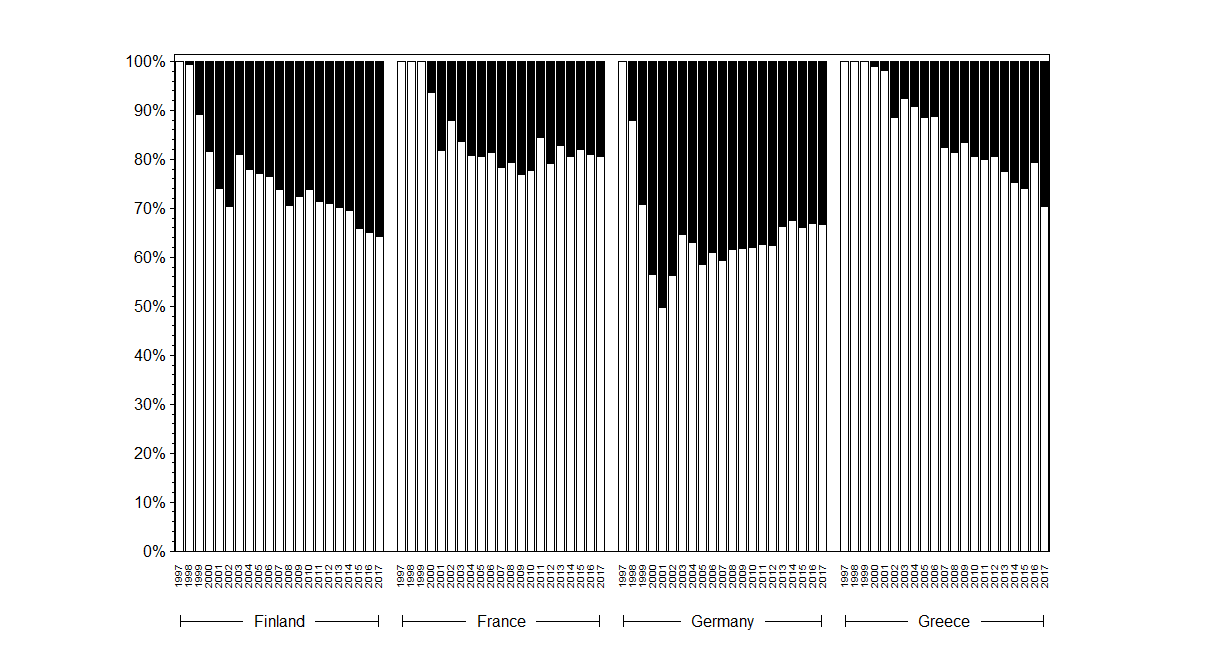
**

= levofloxacin and moxifloxacin, = other quinolones

**Figure S5.** *Continued*

**
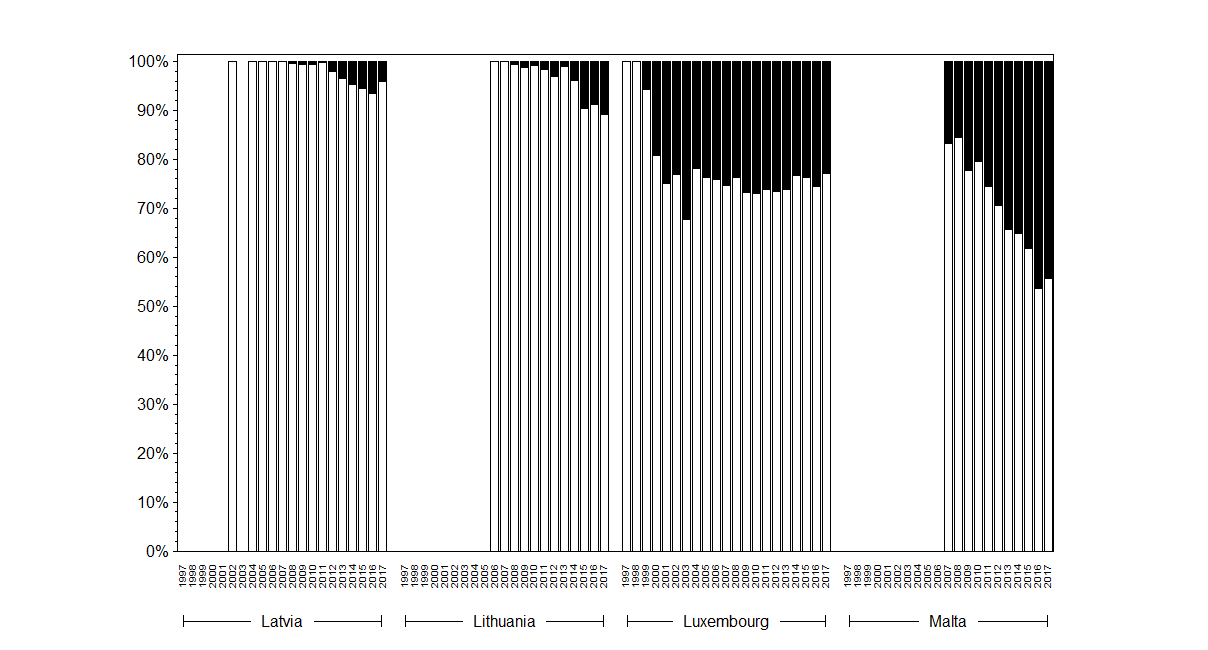

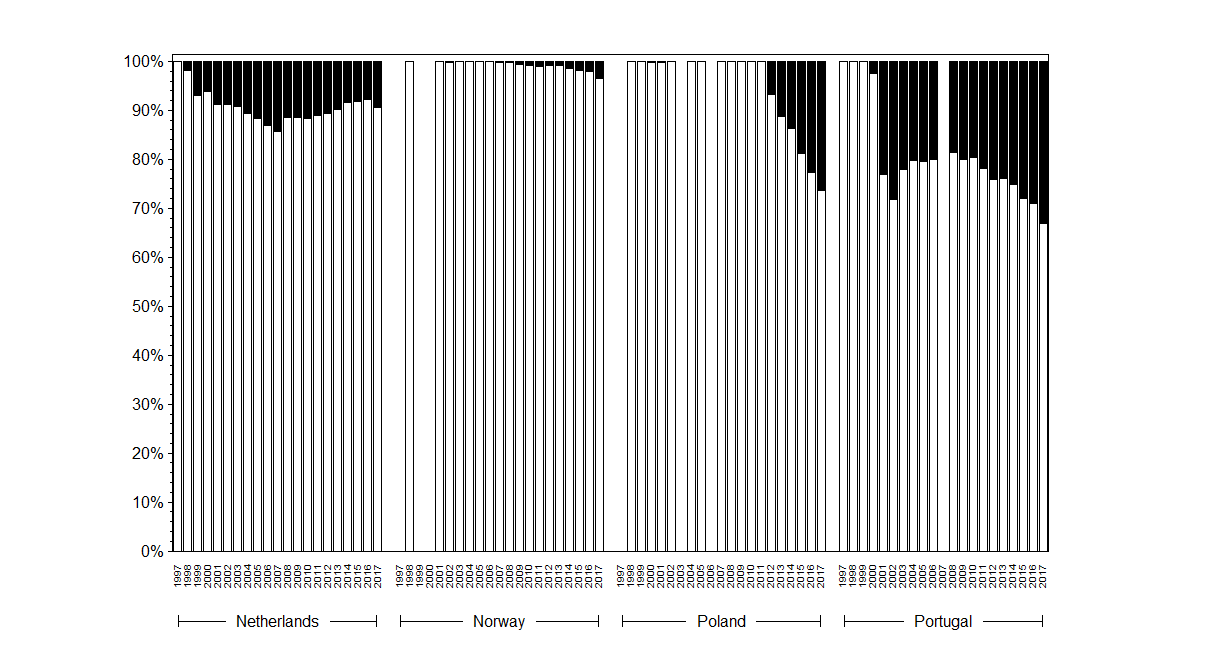
**

= levofloxacin and moxifloxacin, = other quinolones

**Figure S5.** *Continued*

**
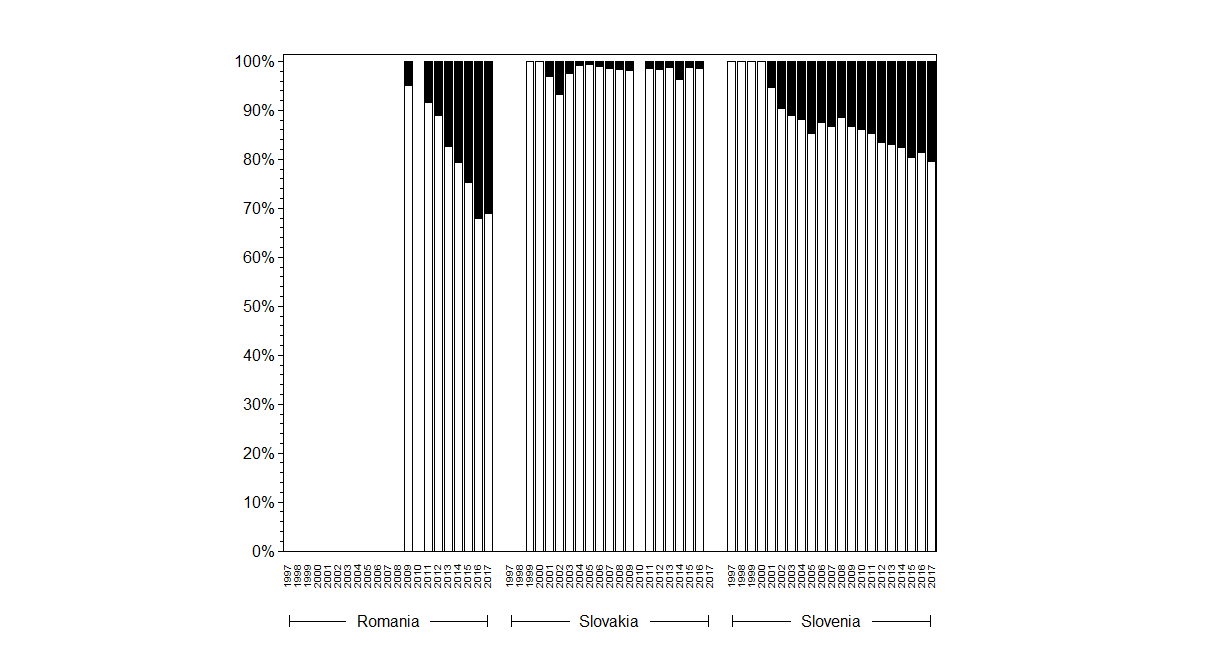
**


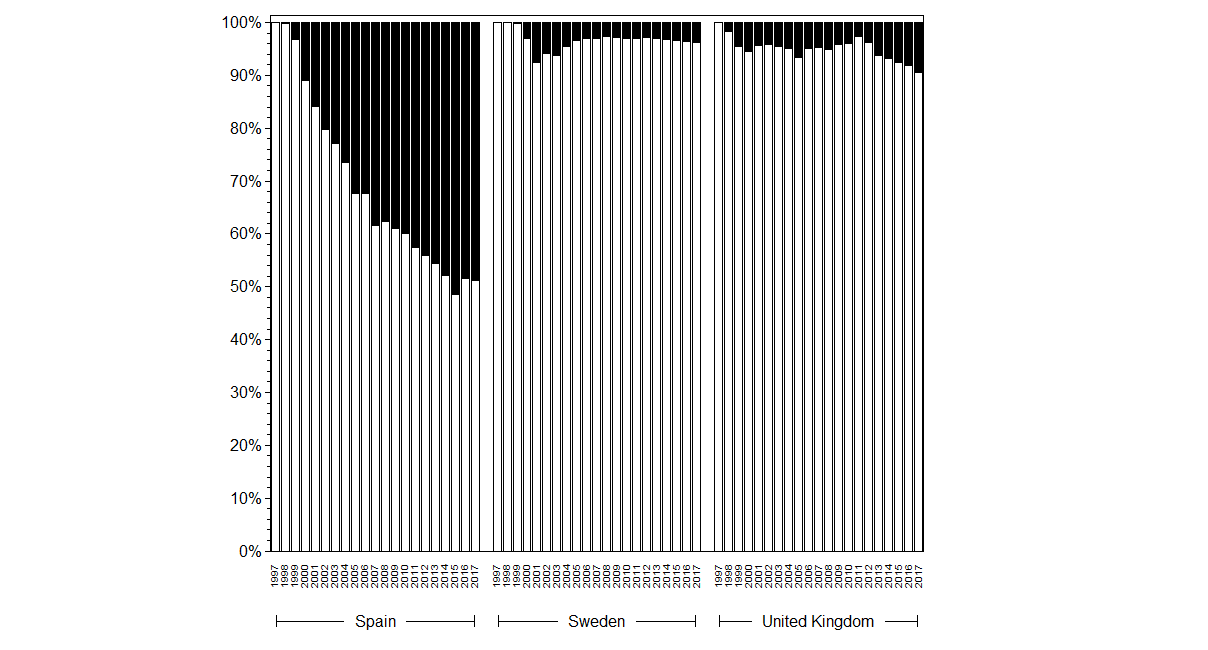


= levofloxacin and moxifloxacin, = other quinolones

**Figure S5.** *Continued*
